# Fibroblastic reticular cell response to dendritic cells requires coordinated activity of podoplanin, CD44 and CD9

**DOI:** 10.1242/jcs.258610

**Published:** 2021-07-22

**Authors:** Charlotte M. de Winde, Spyridon Makris, Lindsey J. Millward, Jesús A. Cantoral-Rebordinos, Agnesska C. Benjamin, Víctor G. Martínez, Sophie E. Acton

**Affiliations:** Stromal Immunology Group, MRC Laboratory for Molecular Cell Biology, University College London, Gower Street, London WC1E 6BT, UK

**Keywords:** Fibroblastic reticular cell, Lymph node, Podoplanin, CD44, CD9, Tetraspanins, Dendritic cell

## Abstract

In adaptive immunity, CLEC-2^+^ dendritic cells (DCs) contact fibroblastic reticular cells (FRCs) inhibiting podoplanin-dependent actomyosin contractility, permitting FRC spreading and lymph node expansion. The molecular mechanisms controlling lymph node remodelling are incompletely understood. We asked how podoplanin is regulated on FRCs in the early phase of lymph node expansion, and which other proteins are required for the FRC response to DCs. We find that podoplanin and its partner proteins CD44 and CD9 are differentially expressed by specific lymph node stromal populations *in vivo*, and their expression in FRCs is coregulated by CLEC-2 (encoded by *CLEC1B*). Both CD44 and CD9 suppress podoplanin-dependent contractility. We find that beyond contractility, podoplanin is required for FRC polarity and alignment. Independently of podoplanin, CD44 and CD9 affect FRC–FRC interactions. Furthermore, our data show that remodelling of the FRC cytoskeleton in response to DCs is a two-step process requiring podoplanin partner proteins CD44 and CD9. Firstly, CLEC-2 and podoplanin binding inhibits FRC contractility, and, secondly, FRCs form protrusions and spread, which requires both CD44 and CD9. Together, we show a multi-faceted FRC response to DCs, which requires CD44 and CD9 in addition to podoplanin.

## Abstract (precis)

**Summary:** There is a multi-faceted response of lymph node fibroblasts to dendritic cells in an adaptive immune reaction, which requires the coordinated activity of partner proteins podoplanin, CD44 and CD9.

## INTRODUCTION

During the adaptive immune response, the lymph node rapidly expands to accommodate the increased number of proliferating lymphocytes (Acton et al., 2014; Astarita et al., 2015; Yang et al., 2014). Effective immune responses require lymph node remodelling at speed, while maintaining tissue architecture, but it is equally important that remodelling is reversible and that tissue damage is prevented.

The lymph node is a highly structured organ consisting of different functional zones, which are organised by a connected network of fibroblastic reticular cells (FRCs) (Fletcher et al., 2015). T-cell zone FRCs (TRCs) must endure and adapt to the pressure of expanding T-cell populations rapidly requiring additional space (Acton et al., 2014; Astarita et al., 2015; Kumar et al., 2015; Yang et al., 2014). Throughout expansion, the FRC network remains connected (Acton et al., 2014; Yang et al., 2014) and aligned with extracellular matrix structures (Astarita et al., 2015; Martinez et al., 2019). Initially, FRCs elongate to stretch the existing network, before proliferating to expand the lymph node further (Acton et al., 2014; Astarita et al., 2015; Yang et al., 2014). Migratory dendritic cells (DCs), expressing the C-type lectin-like receptor CLEC-2 (encoded by *CLEC1B*), are required to initiate FRC network remodelling (Acton et al., 2014; Astarita et al., 2015). When CLEC-2 expression is deleted in DCs, lymph nodes fail to expand relative to controls and the FRC network becomes disrupted (Acton et al., 2014; Astarita et al., 2015). CLEC-2 interacts with the glycoprotein podoplanin on the FRC network (Acton et al., 2012, 2014; Astarita et al., 2015). Podoplanin connects the cytoskeleton to the cell membrane through ezrin-radixin-moesin (ERM) protein binding and drives activation of RhoA and RhoC (RhoA/C) (Acton et al., 2014; Astarita et al., 2015; Martín-Villar et al., 2006). CLEC-2^+^ migratory DCs bind and cluster podoplanin, uncoupling podoplanin from RhoA/C activity, and permitting rapid lymph node expansion (Acton et al., 2014; Astarita et al., 2015). However, the molecular role of podoplanin on FRCs, and its requirement for FRCs to respond to CLEC-2^+^ migratory DCs, is incompletely understood.

Podoplanin was discovered almost simultaneously in a wide variety of tissues and cell types, and has therefore been assigned multiple names (podoplanin, gp38, Aggrus, PA2.26, D2-40 and T1α) based on its function in different contexts (Quintanilla et al., 2019). Podoplanin is widely expressed and plays a pivotal role in the correct development of heart, lungs, secondary lymphoid tissues and lymphatic vasculature. Podoplanin-null mice exhibit embryonic lethality due to cardiovascular problems or die shortly after birth of respiratory failure (Mahtab et al., 2008, 2009; Ramirez et al., 2003), and exhibit defective blood-lymphatic vasculature separation (Schacht et al., 2003; Suzuki-Inoue et al., 2010). Podoplanin expression by FRCs is essential for lymph node development, and the maintenance of high-endothelial venule function via interactions with CLEC-2-expressing platelets (Bénézech et al., 2014; Herzog et al., 2013). Podoplanin has a very short cytoplasmic tail (Martín-Villar et al., 2005), and the two serine residues in this tail (S167 and S171) can be phosphorylated, which regulates cell motility (Krishnan et al., 2013). The cytoplasmic tail of podoplanin consists of only nine amino acids (Martín-Villar et al., 2005), therefore it is suggested that podoplanin requires partner proteins to execute its functions. Many partner proteins have already been identified (Astarita et al., 2012; Quintanilla et al., 2019), but their functions in lymph node remodelling have not been addressed.

Here, we found that two known podoplanin partner proteins, the hyaluronan receptor CD44 (Martín-Villar et al., 2010; Montero-Montero et al., 2020) and tetraspanin CD9 (Nakazawa et al., 2008), are transcriptionally regulated in response to CLEC-2, and their expression is controlled on the surface of FRC populations *in vivo* during an immune response. In other biological contexts, CD44 and CD9 link extracellular cues to intracellular signalling. Tetraspanins are a superfamily of four-transmembrane proteins that form tetraspanin-enriched microdomains via interactions with each other and partner proteins. These microdomains spatially organize the plasma membrane into a tetraspanin web, which facilitates cellular communication (Termini and Gillette, 2017; van Deventer et al., 2017). The interaction of podoplanin with the tetraspanin CD9 is mediated by CD9 transmembrane domains 1 and 2, and this interaction impairs cancer metastasis by inhibiting platelet aggregation (Nakazawa et al., 2008). This is suggestive of a functional role in CLEC-2/podoplanin signalling, since platelets express high levels of CLEC-2. CD9 also controls cell fusion and adhesion (Jiang et al., 2015; Kaji et al., 2000; Reyes et al., 2018). To date, no function of CD9 has been assigned to FRC biology.

Podoplanin interacts with CD44 through their transmembrane domains, and this interaction is modulated by both the cytosolic and transmembrane regions (Montero-Montero et al., 2020). The podoplanin–CD44 interaction at tumour cell protrusions promotes cancer cell migration (Martín-Villar et al., 2010). Similar to what is seen for podoplanin, CD44 also binds ERM proteins (Tsukita et al., 1994). Interestingly, in NIH/3T3 fibroblasts, co-expression of CD44 and podoplanin reversed the hypercontractile phenotype seen in cells overexpressing podoplanin (Acton et al., 2014), suggesting an inhibitory function for CD44 in driving actomyosin contractility in fibroblasts. It has previously been shown that podoplanin and CD44 both reside in cholesterol-rich membrane regions on MDCK cells (Fernández-Muñoz et al., 2011). CLEC-2 binding to FRCs drives podoplanin clustering into cholesterol-rich domains (Acton et al., 2014), but the function of these podoplanin clusters is unknown.

In this study, we seek to further our understanding on the role of podoplanin and its partner proteins in FRC function during lymph node expansion. We investigate the functions of CD44 and CD9 in the response of FRCs to CLEC-2^+^ DCs, the critical initiating step for FRC network spreading and lymph node expansion. We also investigate the requirement for podoplanin signalling with these partner proteins to control FRC functions beyond actomyosin contractility.

## RESULTS

### Increased expression of podoplanin and CD44 on TRCs during adaptive immune responses

It is known that FRC numbers remain constant in the first acute phase of lymph node expansion, and that FRC proliferation is induced later during tissue remodelling (Acton et al., 2014; Astarita et al., 2015; Yang et al., 2014). In the acute phase, CLEC-2 expressed on DCs inhibits podoplanin-driven actomyosin contractility in FRCs (Acton et al., 2014; Astarita et al., 2015), allowing the FRC network to elongate to accommodate the increased number of lymphocytes (Acton et al., 2014; Astarita et al., 2015; Yang et al., 2014). However, the molecular mechanisms are still incompletely understood.

**Fig. 1.**
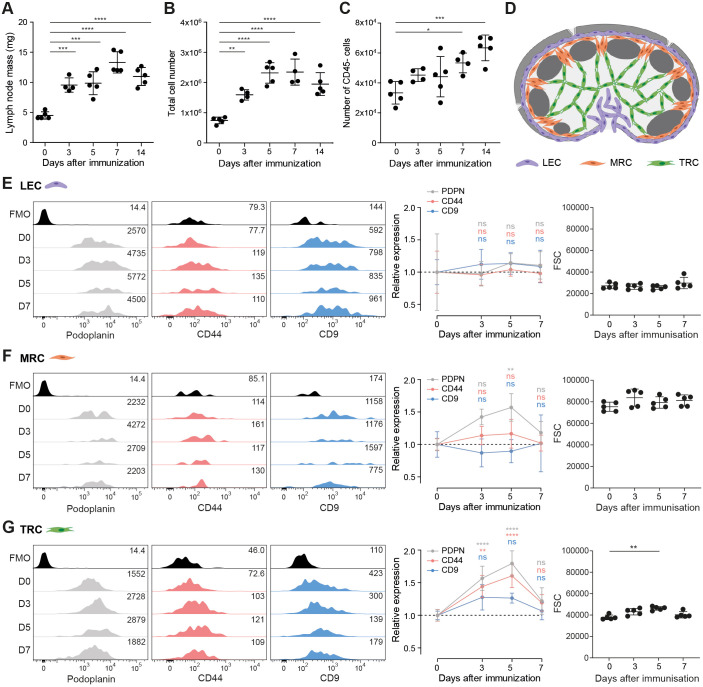
**Increased expression of podoplanin and CD44 on TRCs upon *in vivo* immunization.** (A) Mass (mg) of dissected inguinal lymph nodes. (B,C,E–G) Analysis by flow cytometry of cell suspensions of inguinal lymph nodes from C57BL/6 mice immunized with IFA/OVA for indicated time points. FRC population is based on total lymph node cell count×(percentage of live cells)×(percentage of CD45^−^ cells). Gating strategy is included in Fig. S1A. (B) Total live-cell number based on the live-cell gate as measured by flow cytometry. (C) Number of CD45^−^ cells. (D) Schematic representation of the location of different lymph node stromal cell subsets. Lymphatic endothelial cell (LEC; purple), marginal zone reticular cell (MRC; orange), and T-cell zone reticular cell (TRC; green). (E–G) Podoplanin (PDPN; grey), CD44 (pink) and CD9 (blue) surface expression on LECs (CD31^+^podoplanin^+^ MAdCAM-1^−^; E), MRCs (CD31^−^podoplanin^+^MAdCAM-1^+^; F) and TRCs (CD31^−^podoplanin^+^MAdCAM-1^−^; G) as measured by flow cytometry. Left panels show representative histograms of surface protein expression of podoplanin (grey), CD44 (pink) and CD9 (blue). FMO, fluorescence minus one. Numbers indicate geometric mean fluorescence intensity (gMFI). Middle panels show surface protein expression normalized to day 0 and relative gMFI expression for each marker per cell type is shown. Right panels show forward scatter (FSC) for each lymph node stromal cell subset. Error bars show s.d.; *n*=4–5 mice per time point. *P<0.05; **P<0.01; ***P<0.001; ****P<0.0001; ns, not significant [one-way (A–C) or two-way (E–G) ANOVA with Tukey's multiple comparisons; FSC data (G, right panel) was analysed using Kruskal–Wallis test with Dunn's multiple comparisons].

In agreement with previous reports (Acton et al., 2014; Kumar et al., 2015; Yang et al., 2014), we find that lymph node mass increased 2–3-fold (Fig. 1A) following immunization with an emulsion of incomplete Freund's adjuvant with ovalbumin (IFA/OVA), driven primarily by the increased numbers of lymphocytes (Fig. S1). Despite a rapid increase in lymph node mass (Fig. 1A) and total cellularity (Fig. 1B), the number of CD45^−^ lymph node stromal cells did not significantly increase in the first 5 days post immunization (Fig. 1C). However, stromal cell numbers remained higher than steady state levels, even after lymphocytes had started to traffic out of the tissue (day 7–14) (Fig. 1C; Fig. S1B). We compared the response of lymphatic endothelial cells (LECs; CD31^+^PDPN^+^MAdCAM-1^−^), marginal reticular cells (MRCs; CD31^−^PDPN^+^MAdCAM-1^+^) and T-cell zone FRCs (TRCs; CD31^−^PDPN^+^MAdCAM-1^−^) through the acute phase of lymph node expansion (day 0–7) (Fig. 1D). We here define TRCs to include all CD31^−^PDPN^+^MAdCAM-1^−^ FRC subsets (Huang et al., 2018; Rodda et al., 2018; Sitnik et al., 2016). We found that surface expression of podoplanin did not change on LECs (Fig. 1E), but was increased in both MRCs and TRCs (Fig. 1F,G), as has been reported in other *in vivo* immunization models (Kumar et al., 2015; Yang et al., 2014). However, the increased podoplanin expression on FRCs upon immunization is counterintuitive, since podoplanin drives FRC contractility, which is inhibited during this early phase of lymph node expansion by CLEC-2^+^ DCs.

Since podoplanin is induced to cluster in cholesterol-rich regions upon CLEC-2 binding (Acton et al., 2014), we hypothesised that membrane partner proteins of podoplanin may also be required to downregulate podoplanin-driven contractility for FRC network elongation. A known partner protein of podoplanin, the hyaluronic acid receptor CD44 (Martín-Villar et al., 2010; Montero-Montero et al., 2020), was also specifically upregulated on TRCs, but not on MRCs or LECs (Fig. 1E–G). The levels of another podoplanin partner protein, tetraspanin CD9 (Nakazawa et al., 2008), did not significantly change on any of the lymphoid stromal cell subtypes (Fig. 1E–G), but there was a trend towards higher expression on TRCs (Fig. 1G). Increase in surface expression can be protein upregulation or due to increase in cell size. To address this, we analysed the forward scatter (FSC) as a proxy for cell size (Fig. 1E–G). Although TRC size did increase at day 5 after immunization as previously reported (Acton et al., 2014; Yang et al., 2014), this was not to the same extent as the increase in surface expression of podoplanin and CD44 (Fig. 1E–G).

TRCs are required to elongate upon immune activation, permitting space for rapidly increasing T-cell populations (Acton et al., 2014; Yang et al., 2014). Since podoplanin levels are increased during a phase of lymph node expansion when TRCs are less contractile, we hypothesise that podoplanin may play additional roles on TRCs during an immune response. Interactions with partner proteins CD44 and potentially CD9 may facilitate alternative and unreported podoplanin functions.

### Expression of podoplanin, CD44 and CD9 on FRCs is coregulated by CLEC-2

Investigating the roles of podoplanin, CD44 and CD9 specifically on TRCs *in vivo* is technically challenging since these proteins are broadly expressed in many cell types and carry out essential functions in development and homeostasis. To investigate the functions of podoplanin, CD44 and CD9 in TRCs, we utilised an immortalised FRC cell line (Acton et al., 2014). We can model the TRC responses during acute lymph node expansion by exposing the FRC cell line to recombinant CLEC-2, or model the more prolonged CLEC-2 exposure from migratory DCs arriving into the lymph node over several days (Acton et al., 2014; Astarita et al., 2015) using a CLEC-2-Fc-secreting FRC cell line (Martinez et al., 2019).

**Fig. 2.**
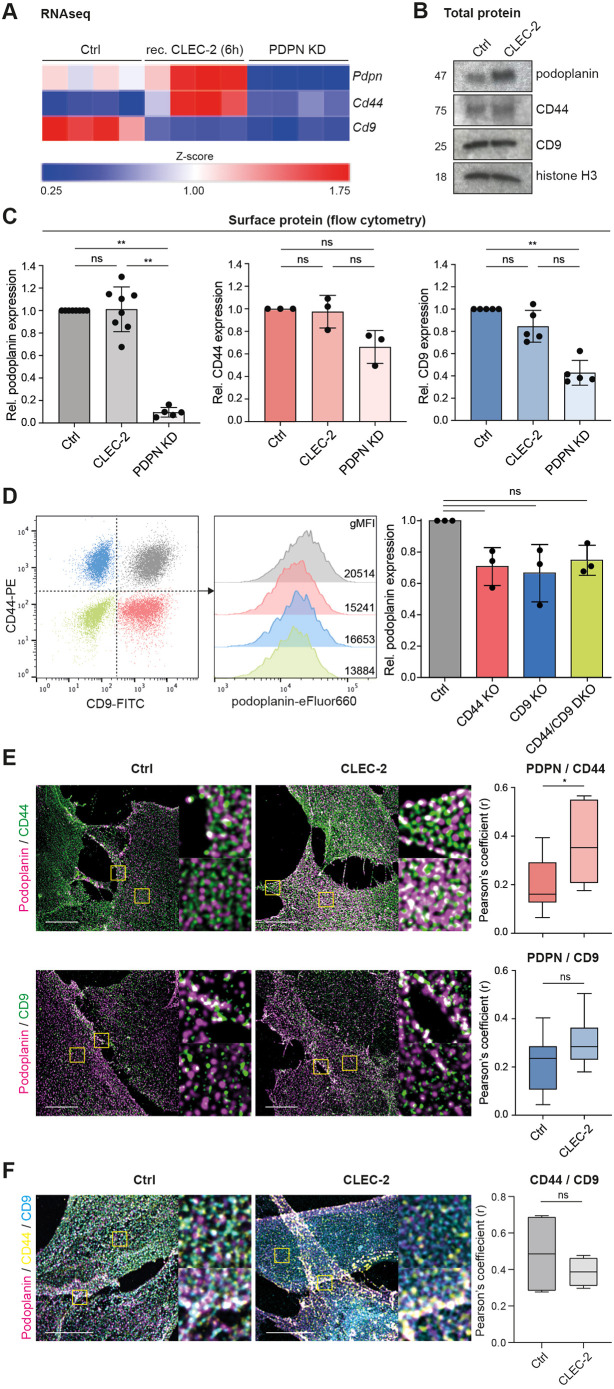
**Expression of podoplanin, CD44 and CD9 on FRCs is coregulated by CLEC-2.** (A) Analysis of RNA-seq expression (Martinez et al., 2019) of *Pdpn* (top row), *Cd44* (middle row) and *Cd9* (bottom row) in unstimulated (left), or short-term stimulated (6 h recombinant CLEC-2-Fc), control (Ctrl; middle), or podoplanin shRNA knock-down (PDPN KD) (right) FRC cell lines. mRNA expression is analysed as *Z*-score compared to *Pdpn* expression in unstimulated Ctrl FRCs. *n*=4 biological replicates per cell type or stimulation. (B) Western blot analysis of total podoplanin, CD44 and CD9 expression in Ctrl or CLEC-2-Fc-expressing FRC cell lines. Histone H3 is used as loading control. Representative data from *n*=3 biological replicates is shown. (C) Podoplanin (left), CD44 (middle) and CD9 (right) surface expression on control (Ctrl; dark), CLEC-2-expressing (CLEC-2; lighter) and PDPN KD (light) FRC cell lines as determined by flow cytometry. Protein expression is normalized to the level in Ctrl FRCs for each individual experiment. Data shown as mean±s.d. with dots representing biological replicates (*n*=3–8). **P<0.01; ns, not significant (Kruskal–Wallis test with Dunn's multiple comparisons). (D) Podoplanin surface expression on indicated FRC cell lines by flow cytometry. Podoplanin expression is normalized to the level in Ctrl FRCs for each individual experiment. Data shown as mean±s.d. with dots representing biological replicates (*n*=3). ns, not significant (Kruskal–Wallis test with Dunn's multiple comparisons). (E) Left panel, double immunofluorescence staining of podoplanin (magenta) and CD44 (top; green) or CD9 (bottom; green) in control (Ctrl) or CLEC-2-expressing (CLEC-2) FRC cell lines. Maximum *Z*-stack projections of representative images are shown. Scale bars: 10 μm. Yellow boxes indicate areas shown in the magnifications. Right panel, colocalisation of podoplanin with CD44 (red) or CD9 (blue) in Ctrl and CLEC-2 FRCs as measured by Pearson's coefficient (*r*) in single sections using JaCoP plugin in Fiji/ImageJ. Data shown as box plots where box represents the 25–75th percentiles, and the median is indicated. The whiskers show the range (*n*=6–7 fields of view per cell line collated from two biological replicates). *P=0.0476; ns, not significant (unpaired two-tailed *t*-test). (F) Left panel, triple immunofluorescence staining of podoplanin (magenta), CD44 (yellow) and CD9 (blue) in control (Ctrl) or CLEC-2-expressing (CLEC-2) FRC cell lines. Maximum *Z*-stack projections of representative images are shown. Scale bars: 10 μm. Yellow boxes indicate areas shown in the magnifications. Right panel, colocalisation of CD44 with CD9 in Ctrl and CLEC-2 FRCs as measured by Pearson's coefficient (*r*) in single sections using JaCoP plugin in Fiji/ImageJ. Data shown as box plots where box represents the 25–75th percentiles, and the median is indicated. The whiskers show the range (*n*=4 fields of view per cell line collated from two biological replicates). ns, not significant (unpaired two-tailed *t*-test).

Both MRCs and TRCs will contact CLEC-2^+^ migratory DCs entering the lymph node (Acton et al., 2012, 2014; Astarita et al., 2015; Katakai, 2012), and both upregulated podoplanin expression within this early timeframe (Fig. 1F,G). We next asked whether the changes in expression of podoplanin, CD44 and CD9 were transcriptionally regulated by CLEC-2. Indeed, we found in previous RNA sequencing (RNA-seq) data (Martinez et al., 2019) that podoplanin (*Pdpn*) mRNA levels are increased upon short-term CLEC-2 stimulation (Fig. 2A). Furthermore, CLEC-2 stimulation *in vitro* also increased *Cd44* mRNA levels (Fig. 2A), which mimicked the increased expression on TRCs during lymph node expansion (Fig. 1G). A podoplanin shRNA knockdown (PDPN KD) FRC cell line (Acton et al., 2014) was used as negative control, and we found that both *Cd44* and *Cd9* expression are reduced when podoplanin expression is knocked down. Using our *in vitro* model system, we showed that CLEC-2 binding is sufficient to increase podoplanin protein expression in an immortalized FRC cell line (Acton et al., 2014; Martinez et al., 2019) (Fig. 2B). However, CLEC-2 alone was not sufficient to change total protein levels of CD44 and CD9 (Fig. 2B). We further validated these findings by measuring surface expression of podoplanin, CD44 and CD9 by flow cytometry using an alternative antibody (Fig. 2C). Podoplanin and CD44 were not increased in the CLEC-2-expressing FRC cell line (Fig. 2C), suggesting that, although more mRNA and total protein is produced (Fig. 2A,B), surface expression is already maximal. The RNA-seq data show a decrease in *Cd9* mRNA expression upon short-term CLEC-2 stimulation (Fig. 2A). In PDPN KD FRCs, both CD44 and CD9 surface protein levels are reduced (Fig. 2A,C), indicating a degree of co-expression between these partner proteins. We investigated this interdependence by generating CD44 and CD9 knockout (KO) FRC cell lines (Fig. S2), and found that knockout of either CD44 or CD9, or both proteins (CD44/CD9 DKO), resulted in an ∼25% reduction of podoplanin surface expression compared to that in control FRCs (Fig. 2D). These data suggest that the availability of these two partner proteins impacts podoplanin expression levels at the plasma membrane, providing additional evidence that podoplanin, CD44 and CD9 are coregulated.

It is reported that podoplanin and CD44 can directly interact via their transmembrane domains (Montero-Montero et al., 2020). Podoplanin and CD9 are also confirmed as direct partner proteins, also interacting through the transmembrane domain of podoplanin and CD9 transmembrane domains 1 and 2 (Nakazawa et al., 2008). We examined colocalization of podoplanin–CD44 and podoplanin–CD9 complexes on the plasma membrane of FRC cell lines. In steady-state, unstimulated FRCs, both podoplanin–CD44 and podoplanin–CD9 complexes partially colocalised, predominantly at the cell periphery (Fig. 2E). CLEC-2-expressing FRCs showed significantly increased colocalization of podoplanin–CD44 broadly across the whole cell membrane (Fig. 2E). The abundance of podoplanin–CD9 complexes was not significantly altered by CLEC-2 stimulation and complexes remained localised to the cell periphery. Thus, upon CLEC-2 stimulation, podoplanin–CD44 and podoplanin–CD9 complexes reside in different subcellular locations, supporting a model of distinct pools of podoplanin on the FRC cell membrane, which may have different functions.

Next, we performed triple staining to further investigate different pools of podoplanin and its partner proteins (Fig. 2F). In control FRC cells and following CLEC-2 treatment, we observed co-localisation of all three proteins (as white dots) raising the possibility of a ternary complex of podoplanin, CD44 and CD9 (Fig. 2F). In line with this, we found that CD44 and CD9 were not expressed in completely separate membrane regions, but did not find any change to CD44 and CD9 colocalization in the presence of CLEC-2 (Fig. 2F). *In vivo*, TRCs showed heterogenous expression of CD44 and CD9 (Fig. S1C). However, comparing expression at steady-state with day 5 following immunogenic challenge, showed a clear correlation in upregulated expression of CD44 and CD9 at the single-cell level (Fig. S1C).

We show that CLEC-2 stimulation of FRCs is sufficient to regulate expression of podoplanin, CD44 and CD9 mRNA and protein, and furthermore, that this treatment regulates their colocalization at the cell membrane. Our data suggest that contact between CLEC-2^+^ DCs and lymphoid fibroblasts would be sufficient to regulate the expression of these surface markers *in vivo* (Fig. 1F,G).

### CD44 and CD9 balance podoplanin-driven FRC contractility

Podoplanin drives actomyosin contractility in FRCs (Acton et al., 2014; Astarita et al., 2015). It has been shown that podoplanin-mediated contractility can be counterbalanced by CD44 overexpression (Acton et al., 2014; Martín-Villar et al., 2010), which coincides with podoplanin re-localising to cholesterol-rich membrane domains (Acton et al., 2014). Indeed, cholesterol depletion in FRCs results in hypercontractility and cell rounding in a podoplanin-dependent manner (Acton et al., 2014). Tetraspanins are predicted to have an intramembrane cholesterol-binding pocket controlling their activity (Zimmerman et al., 2016). We hypothesise that podoplanin activity in FRCs is altered through changing microdomain location in the plasma membrane, mediated by its membrane partner proteins CD44 and CD9.

**Fig. 3.**
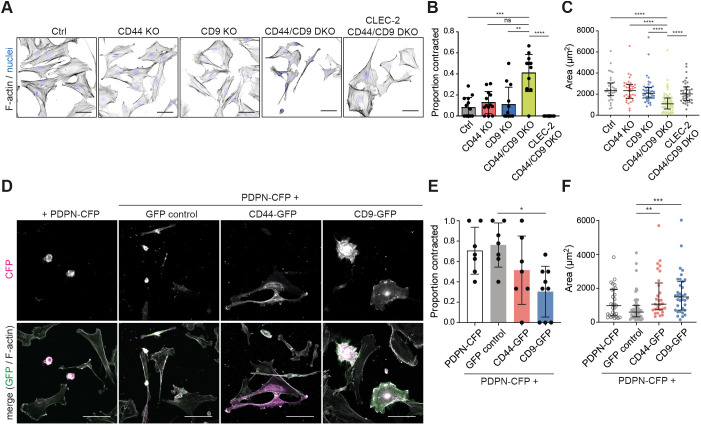
**CD44 and CD9 balance podoplanin-mediated FRC hypercontractility.** (A) Immunofluorescence of F-actin (black) and cell nuclei (blue) in indicated FRC cell line cultures. Maximum *Z*-stack projections of representative images are shown. Scale bars: 50 μm. (B) Proportion of contracted cells in indicated FRC cell line cultures. Data shown as mean±s.d. with dots representing *n*=11–15 images per cell line collated from three or four biological replicates. **P=0.0023; ***P=0.0009; ****P<0.0001; ns, not significant (*P*=0.0528) (Kruskal–Wallis test with Dunn's multiple comparisons). (C) Area (µm^2^) of indicated FRC cell lines. Data shown as median with interquartile range with dots representing individual cells from three or four biological replicates. ****P<0.0001 (Kruskal–Wallis test with Dunn's multiple comparisons). (D) Immunofluorescence of CFP (magenta), GFP (green) and F-actin (white) in the control (Ctrl) FRC cell line transfected with PDPN–CFP alone, or co-transfected with GFP control, CD44–GFP or CD9–GFP. Maximum *Z*-stack projections of representative images are shown. Scale bars: 80 μm. (E) Proportion of contracted cells in the Ctrl FRC cell line transfected with PDPN–CFP alone (white), or co-transfected with GFP control (grey), CD44–GFP (red) or CD9–GFP (blue). Data shown as mean±s.d. with dots representing *n*=7–9 images per cell line collated from three or four biological replicates. *P=0.0186 (Kruskal–Wallis test with Dunn's multiple comparisons). (F) Area (µm^2^) of Ctrl FRCs transfected with PDPN–CFP alone (white), or co-transfected with GFP control (grey), CD44–GFP (red) or CD9–GFP (blue). Data shown as median with interquartile range with dots representing individual cells from three biological replicates. **P=0.0062, ***P=0.0003 (Kruskal–Wallis test with Dunn's multiple comparisons).

The reported function of podoplanin in FRCs is promoting actomyosin contractility (Acton et al., 2014; Astarita et al., 2015). Excess contractility can cause cells to round up, overcoming adhesions, and, in extreme cases, result in membrane blebbing phenotypes (Pinner and Sahai, 2008). CLEC-2 upregulates podoplanin in FRCs (Fig. 2A,B), yet actomyosin contractility is reduced. We tested the capacity for CD44 or CD9 to control podoplanin-driven contractility of FRC cell lines. In the absence of either CD44 or CD9, FRCs remained spread and exhibited F-actin stress fibres, similar to control cells (Fig. 3A,B), indicating that a balance between contraction, protrusion and adhesion is maintained. However, CD44/CD9 DKO cells rounded up and contracted (40% of cells) (Fig. 3A,B), quantified as an ∼50% reduction in cell area (Fig. 3C). CLEC-2 inhibited FRC contractility and induced cell spreading in a podoplanin-dependent manner (Fig. S3). Hypercontractility in CD44/CD9 DKO FRCs was also podoplanin-dependent, since CLEC-2-expressing CD44/CD9 DKO FRCs remained spread (Fig. 3A–C). CD44/CD9 DKO cells expressed lower podoplanin levels (Fig. 2D) yet contracted more than control cells (Fig. 3B), suggesting that both CD44 and CD9 can temper podoplanin-driven contractility. We tested this hypothesis directly by overexpressing PDPN–CFP in combination with CD44 or CD9 GFP fusion proteins in a control FRC cell line (Fig. 3D). Overexpression of PDPN–CFP alone induced control FRCs to round up and cells exhibited membrane blebs (Fig. 3D). Co-transfecting CD44–GFP or CD9–GFP, but not a GFP control plasmid, rescued this hypercontractile phenotype, and FRCs overexpressing both podoplanin and CD44 or CD9 remained spread (Fig. 3D–F). These data indicate that control of podoplanin-driven FRC contractility requires a balanced expression between podoplanin and its partner proteins CD44 and CD9.

### FRC motility and polarity are controlled by podoplanin

We show that podoplanin-driven contractility is reduced by the availability of CD44 and CD9 (Fig. 3). However, these data do not explain why podoplanin expression is upregulated in TRCs during acute lymph node expansion (Fig. 1), a phase of tissue remodelling when the fibroblastic reticular network is elongating and is less contractile (Acton et al., 2014; Astarita et al., 2015; Yang et al., 2014). We asked whether podoplanin controls additional aspects of fibroblastic reticular network function, beyond contractility. To maintain network integrity during acute lymph node expansion, FRCs elongate, reduce adhesion and detach from the underlying conduit network (Martinez et al., 2019). To accommodate the expanding lymphocytes, the FRC network must be flexible and motile, yet retain network connectivity (Acton et al., 2014; Astarita et al., 2015; Martinez et al., 2019; Yang et al., 2014). We tested the requirement for podoplanin, CD44 and CD9 in FRC motility and polarity.

**Fig. 4.**
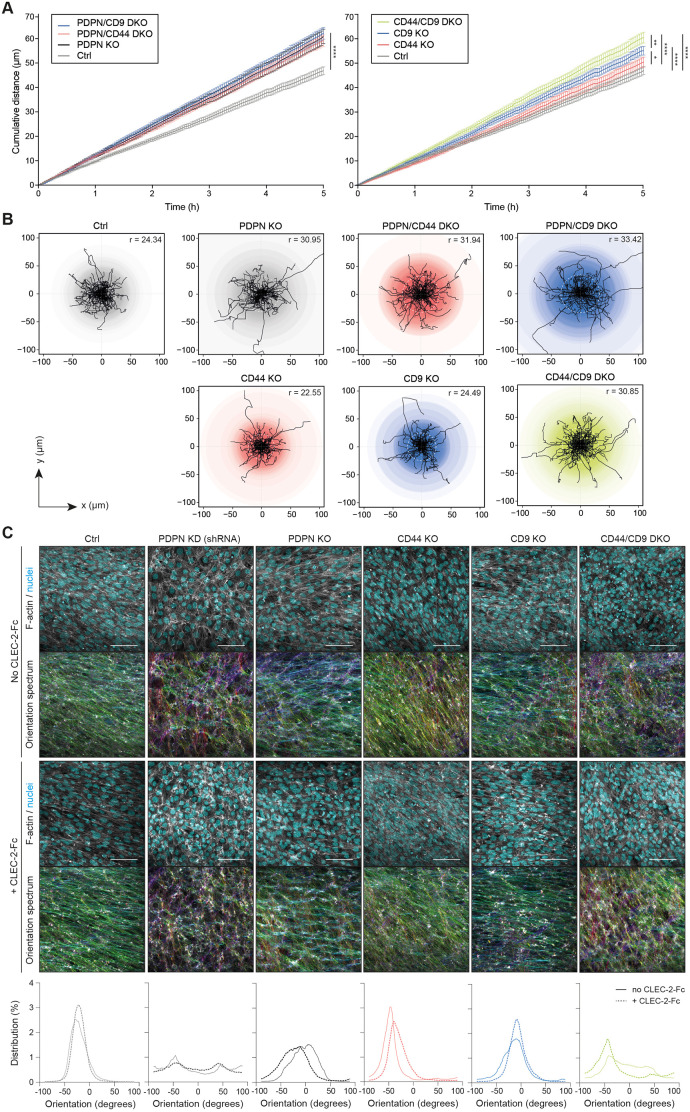
**Podoplanin also controls FRC motility and polarity.** (A) Mean cumulative distance (in µm) travelled by FRC cell lines as indicated in the legend (top left of each graph) over the course of 5 h. Data shown as mean±s.e.m. for each timepoint representing *n*=71–118 cells per cell line. *P<0.05, **P<0.01, ****P<0.0001 (two-way ANOVA with Tukey's multiple comparisons). (B) Individual tracks of FRCs during the first 120 min of the data presented in A. The intensity of the coloured circles represents the number of cells moving the same distance from the starting position. The median maximum distance per condition is depicted in the top right corner and indicated as a dotted white circle in each plot. (C) Top and middle panel. The indicated control (no CLEC-2-Fc) (top) or CLEC-2-Fc-expressing (middle) FRC cell lines were cultured until full confluency was reached, and stained for F-actin (grey) and cell nuclei (cyan). Maximum *Z*-stack projections of representative images from three biological replicates are shown. Scale bars: 100 μm. FRC–FRC alignment was analysed using OrientationJ plugin based on F-actin staining. The colours in the Orientation spectrum indicate the 2D orientation (−180 to 180°) per pixel. Bottom panel, histograms showing the distribution (in percentage) of the pixel orientation (in degrees) per image of no CLEC-2-Fc (solid line) and CLEC-2-Fc-expressing (dashed line) FRC cell lines.

To model FRC motility, we studied the displacement of FRCs in 2D time-lapse assays. It is notable that even as single cells, *in vitro*, control FRCs were not highly migratory, moving at speeds <0.2 µm/min (Fig. 4A,B), which is consistent with their ability to preserve network integrity *in vivo*. PDPN KO FRCs showed increased motility compared to control FRCs, measured by both cumulative distance (Fig. 4A) and displacement (Fig. 4B), which is unaffected by additional knockout of CD44 (PDPN/CD44 DKO) or CD9 (PDPN/CD9 DKO) (Fig. 4A,B). However, in podoplanin^+^ FRCs, both CD44 KO and CD9 KO increased motility in a non-redundant manner (Fig. 4A,B). These data suggest that podoplanin, CD44 and CD9 may have roles to play in maintaining the non-migratory phenotype of FRCs.

Unlike other fibroblast populations, which exhibit contact inhibition of locomotion (CIL) (Park et al., 2020; Roycroft and Mayor, 2016), repolarising and migrating away from neighbouring cells upon contact, FRCs physically connect to form an intricate multicellular network (Acton et al., 2014; Link et al., 2007; Novkovic et al., 2016; Soekarjo et al., 2019; Yang et al., 2014). Network connectivity is maintained and prioritised throughout the early phases of lymph node expansion (Acton et al., 2014; Astarita et al., 2015; Link et al., 2007; Yang et al., 2014). It is unknown how FRCs overcome CIL to form stable connections with their neighbours. Our data show that control podoplanin^+^ FRCs align with each other in *in vitro* cultures; however, both PDPN KD and PDPN KO FRCs lacked this alignment (Fig. 4C). Neither knockout of CD44 nor CD9 altered FRC alignment (Fig. 4C). However, similar to our contractility data (Fig. 3), CD44/CD9 DKO FRCs failed to align (Fig. 4C). Together, these data indicate that podoplanin inhibits FRC motility and promotes alignment, showing for the first time that podoplanin plays an important role in FRC function beyond actomyosin contractility. We then tested the effect of CLEC-2 on FRC alignment, and found that CLEC-2 had no effect on FRC alignment (Fig. 4C), suggesting that cell alignment is cell intrinsic.

### CD9 and CD44 modulate FRC-FRC interactions

Next, we wanted to investigate FRC–FRC interactions in more detail. We noticed a 25% increase in cumulative displacement in CD9 KO FRCs compared to control and CD44 KO FRCs (Fig. 4A), suggesting an independent role for CD9 on FRC motility. In other cell types, CD9 is known to be important for cell migration and adhesion, and cell fusion (Jiang et al., 2015; Kaji et al., 2000; Reyes et al., 2018). Therefore, we further investigated the role for CD9 in FRC motility and FRC-FRC interactions.

**Fig. 5.**
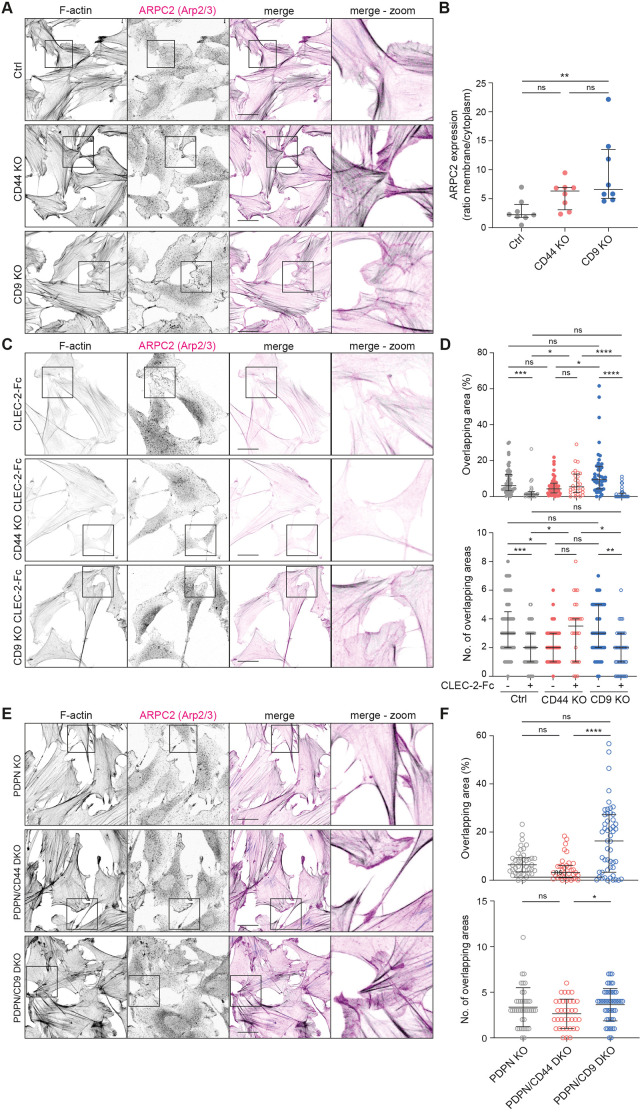
**CD9 and CD44 regulate FRC–FRC interactions.** (A,C,E) Immunofluorescence of F-actin (black) and Arp2/3^+^ protrusions (visualized by staining the ARPC2 subunit; magenta) in indicated FRC cell line cultures. Maximum *Z*-stack projections of representative images from two biological replicates are shown. Scale bars: 30 μm. (B) ARPC2 expression as a ratio of membrane versus cytoplasmic staining in control (Ctrl), CD44 KO and CD9 KO FRC cell lines. Per cell, three line plots were measured and expression ratio was averaged. Only cells with protrusions not touching other cells were analysed. Representative data shown from two biological replicates as median with interquartile range for *n*=8 cells per cell line. (D,F) Percentage of total overlapping area and number of overlapping areas for indicated FRCs. Dots represent single FRCs. *n*=36-57 cells in total from two biological replicates. Error bars represent median with interquartile range. *P<0.05; **P<0.01; ***P<0.001; ****P<0.0001; ns, not significant (Kruskal–Wallis test with Dunn's multiple comparisons).

We compared Arp2/3^+^ protrusions, as a readout of actively protruding plasma membrane (Rottner and Schaks, 2019), in control, CD44 KO and CD9 KO FRC cell lines. FRCs lacking CD9 showed increased membrane localisation of ARPC2, part of the Arp2/3 complex, compared to control or CD44 KO FRCs (Fig. 5A,B). CD9 KO FRCs have broad ARPC2^+^ protrusions, covering most of the plasma membrane and exhibiting continued protrusion and spreading, as such overlapping neighbouring cells (Fig. 5A). Control FRCs meet and interact with their neighbouring FRCs forming connections between one another with little overlap of membranes (Fig. 5A). FRC–FRC connections are important *in vivo* to maintain network integrity. Therefore, we next asked how CLEC-2 affects the interactions between neighbouring FRCs (Fig. 5C,D). CLEC-2 caused FRCs to space out of each other as measured by percentage and number of overlapping areas (Fig. 5C,D). CD9 KO FRCs responded similarly to control FRCs; however, interestingly, CD44 KO FRCs did not change their interactions with neighbouring FRCs in the presence of CLEC-2 (Fig. 5C,D). The effect of CD9 in altering FRC interactions was also observed in PDPN/CD9 DKO cells (Fig. 5E,F), indicating that this phenotype is CD9-dependent and independent of podoplanin. Our data indicate that *in vivo* CD9 may facilitate or modulate FRC network formation at steady state, whereas CD44 is required for modifying FRC–FRC interactions in the presence of CLEC-2^+^ DCs.

### CD44 and CD9 control podoplanin-dependent FRC responses to CLEC-2^+^ DCs

**Fig. 6.**
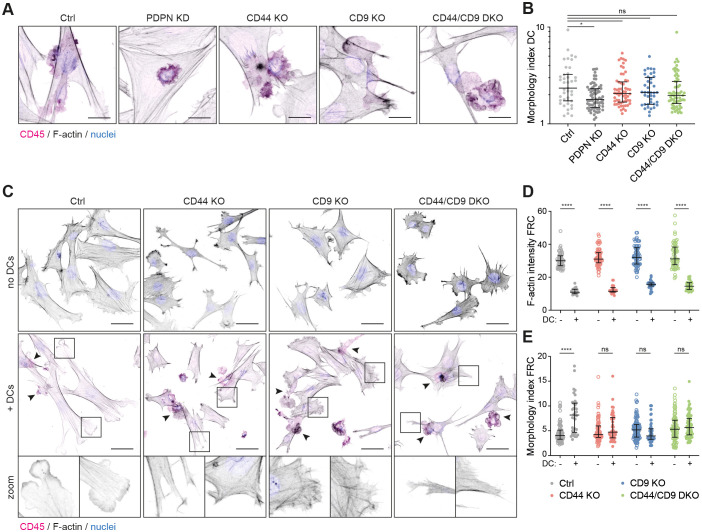
**Podoplanin, CD44 and CD9 are required for FRCs to respond to CLEC-2^+^ DCs.** (A) Immunofluorescence of 3D cultures of indicated FRC cell lines with LPS-stimulated bone marrow-derived DCs (CD45^+^; magenta). Maximum *Z*-stack projections of representative images from *n*=2 biological replicates are shown. Scale bars: 20 μm. (B) Morphology index (perimeter^2^/4π area) of DCs in interaction with an FRC. Dots represent single DCs. *n*=40-72 DCs collated from two biological replicates. Error bars represent median with interquartile range. *P=0.0149; ns, not significant (Kruskal–Wallis test with Dunn's multiple comparisons). The *y*-axis has a log_10_ scale. (C) Immunofluorescence of 3D cultures of FRC cell lines without (upper row) or with (middle and bottom rows) LPS-stimulated bone marrow-derived DCs (magenta). Maximum *Z*-stack projections of representative images from *n*=2 biological replicates are shown. Scale bars: 50 μm. (D,E) F-actin intensity (mean grey value of phalloidin–TRITC staining; D), and morphology index (perimeter^2^/4π area; E) of indicated FRCs alone (open circles) or in interaction with a DC (closed circles). Dots represent single FRCs. *n*=36–104 FRCs collated from two biological replicates. Error bars represent median with interquartile range. ****P<0.0001; ns, not significant (two-way ANOVA with Tukey's multiple comparisons).

Podoplanin was first described as a ligand promoting both platelet aggregation and DC migration (Acton et al., 2012; Suzuki-Inoue et al., 2010). We next tested whether CD44 or CD9 expression by FRCs is required for podoplanin ligand function by performing 3D co-culture of FRC cell lines with bone marrow-derived CLEC-2^+^ DCs. Contact with podoplanin^+^ FRCs induces DCs to extend protrusions, in a CLEC-2 (Acton et al., 2012) and tetraspanin CD37-dependent manner (de Winde et al., 2018). DCs co-cultured with PDPN KD FRCs did not spread or make protrusions (Fig. 6A). However, co-culture of DCs with CD44 KO or CD9 KO FRCs did not hamper DC responses (Fig. 6A). Furthermore, the increase in morphology index (perimeter^2^/4π area) was equivalent to that seen with DCs co-cultured with control FRCs (Fig. 6B). As such, podoplanin ligand function is not dependent on CD44 or CD9 expression on FRCs. This is in agreement with published data showing that soluble recombinant podoplanin-Fc can induce DC protrusions (Acton et al., 2012; de Winde et al., 2018).

In the lymph node, the fibroblastic reticular network is primed to remodel and elongate during immune responses by contact with CLEC-2^+^ DCs (Acton et al., 2014; Astarita et al., 2015). It is known that podoplanin expression by FRCs is required for this signalling pathway, which primarily inhibits actomyosin contractility (Acton et al., 2014; Astarita et al., 2015). Since we have shown that CD44 and CD9 both control FRC contractility in a podoplanin-dependent manner (Fig. 3), and may also play roles in FRC motility and FRC–FRC interactions (Figs 4, 5), we asked whether CD44 and/or CD9 expression is required by FRCs to respond to CLEC-2^+^ DCs. Binding of CLEC-2^+^ DCs to FRCs drives elongation and induction of multiple protrusions, which *in vivo*, is required for acute lymph node expansion during adaptive immune responses (Acton et al., 2014; Astarita et al., 2015). FRCs responded to CLEC-2^+^ DCs *in vitro* by forming lamellipodia-like actin-rich protrusions in multiple directions (Fig. 6C), and by a reduction in F-actin fibres (Fig. 6C,D). We interpret this response as reduced actomyosin contractility, and a concurrent increase in actin polymerisation driving protrusions. This was quantified by increased morphology index (perimeter^2^/4π area; Fig. 6E). Strikingly, both CD44 KO and CD9 KO FRCs failed to form lamellipodia in response to DC contact (Fig. 6C). CD44 KO FRCs exhibited small protrusions with F-actin ‘spikes’, whereas CD9 KO FRCs attempted broader protrusions, but failed to accumulate F-actin at the leading edge (Fig. 6C). These defects are quantified by the lack of increased morphology index in response to CLEC-2^+^ DCs (Fig. 6E). However, we still observed a DC-induced reduction in F-actin fibres in CD44 KO and CD9 KO FRCs, as well as in CD44/CD9 DKO FRCs (Fig. 6C,D), confirming that CLEC-2^+^ DCs still contact the FRCs and can inhibit contractility pathways (Fig. 6A). We conclude that both CD44 and CD9 participate in podoplanin-dependent spreading. Indeed, even before contact with DCs, CD44 KO and CD9 KO FRCs were spread over a smaller area compared to control FRCs (Fig. 6C), suggesting that CD44 and CD9 also act to balance podoplanin-driven contractility and protrusion formation in steady state. These data lead us to conclude that the induction of spreading and the formation of lamellipodia protrusions in response to DC contact is an active podoplanin-dependent process, which requires both CD44 and CD9 in a non-redundant fashion.

Overexpression of podoplanin drives hypercontractility (Acton et al., 2014; Astarita et al., 2015), yet when FRCs are spreading and elongating during the initiation of lymph node expansion, podoplanin expression is increased (Fig. 1). Our data demonstrate that podoplanin is involved in multiple FRC functions beyond actomyosin contractility, and that these functions are controlled by the podoplanin partner proteins CD44 and CD9.

## DISCUSSION

Lymph node expansion is a transient and reversible process, a cycle of controlled tissue remodelling through each adaptive immune response (Fletcher et al., 2015). FRCs shape lymph node architecture (Fletcher et al., 2015), and control the balance between actomyosin contractility and spreading/elongation to determine both lymph node structure and size (Acton et al., 2014; Astarita et al., 2015; Link et al., 2007; Yang et al., 2014). Upon initiation of an adaptive immune response, CLEC-2^+^ migratory DCs inhibit podoplanin-driven FRC contractility to permit lymph node expansion (Acton et al., 2014; Astarita et al., 2015). We now show that podoplanin and its partner proteins CD44 and CD9 play key roles in balancing different FRC functions in steady state and in response to CLEC-2^+^ DCs.

We show that podoplanin expression by FRCs controls functions beyond contractility. During an immune response, proliferating T cells provide a mechanical strain for TRCs (Yang et al., 2014), and TRC elongation and spreading is required to preserve network connectivity and stromal architecture, in advance of their proliferation (Acton et al., 2014; Astarita et al., 2015; Yang et al., 2014). We suggest that podoplanin expression by TRCs is pivotal to their adaptable phenotype. Our data show that podoplanin surface expression increases on TRCs in the first 3–5 days after immunization, a period when contractility through the network is reduced. Mechanisms controlling podoplanin expression in FRCs are incompletely understood. Upregulation and maintenance of podoplanin expression may be mediated by lymphotoxin β-receptor (LTβR) stimulation by DCs (Kumar et al., 2015) and/or altered YAP/TAZ signalling (Choi et al., 2020). Here, we find that CLEC-2 stimulation of FRCs directly upregulates podoplanin mRNA and protein expression. Surface expression of CD44 or CD9, or stimulation with CLEC-2 is able to counterbalance podoplanin-driven contractility. We find that in addition to contractility, podoplanin is required to inhibit lymphoid fibroblast motility and preserve polarity, which would be essential for maintaining FRC network integrity in homeostasis and during lymph node expansion.

In this study, we sought to understand the function of other FRC markers in the normal physiology of immune responses. We find a role for tetraspanin CD9 in controlling FRC–FRC interactions. Tetraspanins are membrane-organizing proteins controlling a variety of cellular processes, including cell–cell interactions, cell migration and signalling events (Termini and Gillette, 2017; van Deventer et al., 2017). FRCs lacking CD9 do not detect neighbouring cells, and spread and grow over each other. In other non-lymphoid tissues, CD9 controls cell migration and adhesion, and is required for cell–cell fusion (Jiang et al., 2015; Kaji et al., 2000; Reyes et al., 2018). We hypothesize that CD9 on FRCs contributes to the formation and preservation of the FRC network. We also found that CD44 surface expression increases on TRCs during the initial phase of lymph node expansion. Furthermore, CLEC-2 stimulation increases colocalisation of podoplanin and CD44 on FRCs. We find that both CD44 and CD9 on FRCs are able to suppress podoplanin-driven contractility, facilitating alternative FRC phenotypes. However, in the presence of CLEC-2, which inhibits podoplanin-driven contractility, colocalization of podoplanin with CD9 remains unaltered, in contrast to podoplanin–CD44 colocalization. Thus, the exact role of CD9 in controlling FRC contractility and the molecular mechanisms involved requires further investigation.

It has been previously reported that podoplanin–CLEC-2 binding is necessary for the lymph node to expand in the early phases of an adaptive immune response (Acton et al., 2014; Astarita et al., 2015). Importantly, we now show that podoplanin expression by FRCs is not sufficient for them to respond to CLEC-2^+^ DCs. FRCs in contact with CLEC-2^+^ DCs respond by reducing F-actin cables, making protrusions in multiple directions and spreading over a larger area. It has been assumed that FRC spreading is an indirect event in response to inhibition of actomyosin contractility (Acton et al., 2014; Astarita et al., 2015). We find that FRCs lacking either CD44 or CD9 expression still reduce F-actin cables in response to DCs; however, they fail to make protrusions and spread. Our data show that CD44 is involved in broad lamellipodial protrusions, and CD9 regulates filopodial-like protrusions. We conclude that both CD44 and CD9 are required for DC-mediated FRC elongation and spreading, and we now identify loss of actomyosin contractility and spreading as two differently regulated, but linked, active processes.

The FRC response to CLEC-2^+^ DCs is multifaceted, and we need to understand more about CLEC-2-driven podoplanin signalling controlling these various functions. We know that both podoplanin and CD44 bind ERM proteins, which couple membrane proteins to the actin cytoskeleton, driving contractility (Acton et al., 2014; Astarita et al., 2015; Martín-Villar et al., 2006; Tsukita et al., 1994). CLEC-2 binding results in ERM dephosphorylation and decoupling from podoplanin (Acton et al., 2014; Astarita et al., 2015). We show that CLEC-2 stimulation results in increased colocalisation of podoplanin and CD44 on FRCs. It is currently unknown whether CLEC-2-mediated clustering of podoplanin and CD44 forces uncoupling of ERM proteins, or whether dephosphorylation and uncoupling of ERM proteins by an unknown mechanism provides space for clustering of podoplanin–CD44 complexes. Moreover, further studies are required to identify the specific cascade of signalling events downstream of the CLEC-2/podoplanin axis facilitating FRC spreading and elongation. Our data suggest that the function of podoplanin and downstream signalling would be determined not only by podoplanin expression level or subcellular localisation, but by the availability of its membrane partner proteins CD44 and CD9. Podoplanin, CD44 and CD9 are broadly expressed and their expression can alter in various pathologies (Quintanilla et al., 2019; Reyes et al., 2018; Senbanjo and Chellaiah, 2017). For example, podoplanin expression on FRCs is altered in lymph node-originated haematological malignancies (Apollonio et al., 2018; Pandey et al., 2017 and our own unpublished data). Our findings may further provide insights as to how the balance of their expression may drive cellular functions in pathological conditions.

This study provides a molecular understanding into how FRC functions are controlled in homeostasis and during lymph node expansion. Interactions between podoplanin and two of its known partner proteins, CD44 and CD9, shape dynamic FRC responses, supporting a model of distinct functional protein domains on the FRC plasma membrane. We hypothesize that during homeostasis, lymph node size remains stable via tonic CLEC-2 signalling provided by resident and migratory DCs, maintaining the balance between contraction and protrusion through the FRC network. As the lymph node expands, this mechanical balance in the TRC network is transiently shifted towards cell elongation and protrusion by an influx of CLEC-2^+^ migratory DCs. Our data indicate that TRCs require expression of CD44 and CD9, in addition to podoplanin, to facilitate this shift.

## MATERIALS AND METHODS

Biological materials generated for this study are available upon request to the corresponding author with an materials transfer agreement (MTA) where appropriate.

### Mice

Wild-type C57BL/6J mice were purchased from Charles River Laboratories. Female mice were used for *in vivo* experiments and were aged 6–10 weeks. Mice were age matched and housed in specific pathogen-free conditions. All animal experiments were reviewed and approved by the Animal and Ethical Review Board (AWERB) within University College London and approved by the UK Home Office in accordance with the Animals (Scientific Procedures) Act 1986 and the ARRIVE guidelines.

### *In vivo* immunizations

Mice were immunized via subcutaneous injection in the right flank with 100 µl of an emulsion of ovalbumin (OVA) in incomplete Freund's adjuvant (IFA) (100 µg OVA per mouse; Hooke Laboratories). Draining inguinal lymph nodes were taken for analysis by flow cytometry. Lymph nodes were digested as previously described (Fletcher et al., 2011), and cells were counted using Precision Count Beads as per the supplier's instructions (Biolegend), and stained for analysis by flow cytometry.

### Cell culture

Control and PDPN KD FRC cell lines (Acton et al., 2014), and CLEC-2-Fc expressing FRCs (Martinez et al., 2019) are previously described. ﻿FRC cell lines have been regularly analysed by flow cytometry for authentication, and were screened by the Cell Services Department at the Francis Crick Institute (London, UK) to rule out contamination.

The PDPN KO FRC cell line was generated using CRISPR/Cas9 editing; a control FRC cell line (Acton et al., 2014) was transfected with pRP[CRISPR]-hCas9-U6>{PDPN gRNA 1} plasmid (constructed and packaged by Vectorbuilder; vector ID: VB160517-1061kpr) using Lipofectamine 2000 transfection reagent (Thermo Fisher Scientific). We performed three rounds of transfection, and subsequently performed magnetic cell sorting (MACS) using MACS LD columns (Miltenyi Biotec), anti-mouse podoplanin-biotin antibody (clone 8.1.1, eBioscience, 13-5381-82), and anti-biotin microbeads (Miltenyi Biotec, 130-090-485) as per the supplier's instructions to sort PDPN KO FRCs by negative selection. Complete knockout of podoplanin expression was confirmed using quantitative RT-PCR, flow cytometry and western blotting.

CD44 KO, CD9 KO, and CD44/CD9 DKO FRCs were generated using CRISPR/Cas9 editing. Control, CLEC-2-expressing or PDPN KO FRCs were transfected using Attractene Transfection Reagent (Qiagen) with one or both of the following plasmids: pRP[CRISPR]-hCas9-U6>{CD44-T3 exon 2} (constructed and packaged by Vectorbuilder; vector ID: VB180119-1369pus), or pRP[CRISPR]-hCas9-U6>{CD9 exon 1} (constructed and packaged by Vectorbuilder; vector ID: VB180119-1305adb). Subsequently, CD44 KO, CD9 KO, and CD44/CD9 DKO FRCs underwent two or three rounds of FACS to obtain a full CD44 and/or CD9 KO FRC cell line, which was confirmed using quantitative RT-PCR, flow cytometry and western blotting.

FRC cell lines were cultured in high-glucose DMEM with GlutaMAX supplement (Gibco, via Thermo Fisher Scientific) supplemented with 10% fetal bovine serum (FBS; Sigma-Aldrich), 1% penicillin-streptomycin (P/S) and 1% insulin-transferrin-selenium (both Gibco, via Thermo Fisher Scientific) at 37°C, 10% CO_2_, and passaged using cell dissociation buffer (Gibco, via Thermo Fisher Scientific).

Bone marrow-derived dendritic cells (BMDCs) were generated by culturing murine bone marrow cell suspensions in RPMI 1640 medium (Gibco, via Thermo Fisher Scientific) supplemented with 10% FBS, 1% P/S and 50 µM 2-mercaptoethanol (Gibco, via Thermo Fisher Scientific), and 20 ng/ml recombinant murine granulocyte-macrophage colony-stimulating factor (mGM-CSF, Peprotech, 315-03), as adapted from previously described protocols (Lutz et al., 1999), at 37°C, 5% CO_2_. On day 6, BMDCs were additionally stimulated with 10 ng/ml lipopolysaccharides from *E. coli* (LPS; Sigma-Aldrich, L4391-1MG) for 24 h.

### DC–FRC co-cultures

FRCs (0.7×10^4^ cells per well) were seeded on 24-well glass-bottomed cell culture plates (MatTek) at 37°C, 10% CO_2_. After 24 h, LPS-stimulated BMDCs (2.5×10^5^ cells per well) were seeded into 3D collagen (type I, rat tail) and Matrigel matrix (both from Corning, via Thermo Fisher Scientific) supplemented with 10% minimum essential medium alpha medium (MEMalpha, Invitrogen, via Thermo Fisher Scientific) and 10% FCS (Greiner Bio-One) on top of the FRCs. Co-cultures were incubated overnight at 37°C, 10% CO_2_. The next day, co-cultures were fixed and stained for analysis by microscopy.

### Flow cytometry

For analysis of lymph nodes from *in vivo* immunizations by flow cytometry, 3×10^6^ cells were incubated with purified rat IgG2b anti-mouse CD16/32 receptor antibody as per supplier's instructions (Mouse BD Fc-block, clone 2.4G2, BD Biosciences, 553141) for 20 min at 4°C. Cells were stained with the following primary mouse antibodies (1:100 dilution) for 30 min at 4°C: CD45-BV750 (clone 30-F11, Biolegend, 103157), CD31-PE-Cy5.5 (clone MEC 13.3, BD Biosciences, 562861), podoplanin-PE (clone 8.1.1, BD Biosciences, 566390), CD44-BV605 (clone IM7, BD Biosciences, 563058), CD9-FITC (clone MZ3, Biolegend, 124808) and MAdCAM-1-BV421 (clone MECA-367, BD Biosciences, 742812). Cells were washed with phosphate-buffered saline (PBS) and stained with Zombie Aqua fixable live-dead kit as per the supplier's instructions (Biolegend, 423101) for 30 min at 4°C. Next, cells were fixed using Biolegend fixation/permeabilization buffer as per the supplier's instructions (Biolegend, 421403). Samples were analysed on BD Symphony A5 equipped with 355 nm, 405 nm, 488 nm, 561 nm and 638 nm lasers. Acquisition was set to 5×10^5^ single, live CD45^+^ cells. FRC cell number was based on their percentage within the CD45^−^ cells.

Single-cell suspensions of FRC cell lines were incubated with FcR blocking reagent (Miltenyi Biotec) as per the supplier's instructions, followed by 30 min staining on ice with the following primary mouse antibodies diluted in PBS supplemented with 0.5% bovine serum albumin (BSA) and 5 mM EDTA: hamster anti-podoplanin-eFluor660 (clone 8.1.1, 1:200, eBioscience, 50-5381-82), rat anti-CD44-PE (clone IM7, 1:50, BD Biosciences, 553134), and/or rat anti-CD9-FITC (clone MZ3, 1:50, Biolegend, 124808). Stained cells were analysed using FACSDiva software and LSR II flow cytometer (both BD Biosciences). All flow cytometry data was analysed using FlowJo Software version 10 (BD Biosciences). Antibodies are checked for specificity against genetic knockout cell lines and shown in the data provided.

### Western blotting

Control or CLEC-2-Fc FRCs were plated in a six-well culture plate (10^5^ cells per well). After 24 h, the culture plate was placed on ice and cells were washed twice with cold PBS. Cells were lysed in 100 µl 4× Laemmli lysis buffer (Bio-Rad) and collected using cell scraper. Samples were separated by reducing 10% SDS-polyacrylamide gel electrophoresis. Western blots were incubated with rat anti-mouse podoplanin (clone 8F11, 1:1000, Acris Antibodies, AM26513AF-N), or mouse anti-histone H3 (1:2000, Abcam, ab24824) as loading control, in PBS supplemented with 1% skim milk powder and 0.2% BSA overnight at 4°C, followed by staining with appropriate HRP-conjugated secondary antibodies (Abcam) for 2 h at room temperature. Western blots were developed using Luminata Crescendo Western HRP substrate (Merck Millipore) and imaged on ImageQuant LAS 4000 mini (GE Healthcare Life Sciences).

### Transient transfection

FRCs were plated on glass coverslips in a six-well culture plate (2.5×10^4^ cells per well) 1 day before transfection with PDPN–CFP and CD44–GFP (Acton et al., 2014), CD9–GFP (a kind gift from Dr Sjoerd van Deventer and Prof. Annemiek van Spriel, Radboudumc, Nijmegen, NL) or GFP control plasmid (0.5 µg DNA per plasmid) using Attractene transfection reagent (Qiagen) as per the supplier's instructions. At 24 h post transfection, FRCs were fixed for analysis of cell contractility by microscopy.

### Immunofluorescence

FRCs were seeded on glass coverslips for 24 h at 37°C, 10% CO_2_. Next, cells were fixed in 3.6% formaldehyde (Sigma-Aldrich; diluted in PBS), and subsequently blocked in 2% BSA in PBS and stained for 1 h at room temperature with the following primary mouse antibodies: hamster anti-podoplanin-eFluor660 (clone 8.1.1, 1:200, eBioscience, 50-5381-82), rat anti-CD44 (clone IM7, 1:200, BD Biosciences, 553131), rat anti-CD9-eFluor450 (clone KMC8, 1:200, eBioscience, 14-0091-82), or rabbit anti-p34-Arc/ARPC2 (Arp2/3, 1:100, Merck, 07-227). This was followed by incubation with appropriate Alexa Fluor-conjugated secondary antibodies (1:500, Invitrogen, via Thermo Fisher Scientific) for 1 h at room temperature. F-actin and cell nuclei were visualized using, respectively, phalloidin–TRITC (P1951-1MG) and DAPI (D9542-1MG; both 1:500 dilution, both from Sigma-Aldrich) incubated for 15 min at room temperature, and coverslips were mounted in Mowiol (Sigma-Aldrich). Cells were imaged on a Leica SP5 or SP8 confocal microscope using, respectively, HCX PL APO and HC PL APO CS2 /1.4 63× oil lenses.

DC–FRC cultures were fixed with AntigenFix (DiaPath, via Solmedia) for 3 h at room temperature, followed by permeabilization and blocking with 2% BSA (Sigma-Aldrich) and 0.2% Triton X-100 in PBS for 2 h at room temperature. Subsequently, BMDCs were stained using rat anti-mouse CD45-AF647 (clone 30-F11, 1:250, Biolegend, 103123), and F-actin and cell nuclei were visualized using, respectively, phalloidin–TRITC (P1951-1MG) and DAPI (D9542-1MG; both 1:500 dilution, both from Sigma-Aldrich). Co-cultures were imaged on a Leica SP5 confocal microscope using HCX PL APO /1.25 40× oil lenses.

Images were analysed using Fiji/ImageJ software. *Z*-stacks (0.5 µm/step) were projected with ImageJ Z Project (maximum projection). Podoplanin–CD44 and podoplanin–CD9 colocalisation was analysed by measuring Pearson's coefficient in single sections using the JACoP plugin (Bolte and Cordelières, 2006). The proportion of contracted cells was quantified by analysing the number of contracted/blebbing cells (based on F-actin staining) compared to total number of cells per field of view. The cell area of FRCs was analysed by manually drawing around the cell shape using F-actin staining. FRC alignment was analysed using OrientationJ plugin (Püspöki et al., 2016). ARPC2 expression was calculated as a ratio of membrane versus cytoplasmic staining. Per FRC, three line plots were measured and expression ratio was averaged. Only FRCs with protrusions not touching other cells were analysed. Overlapping cell area was determined based on ARPC2 and F-actin staining to determine cell periphery and separate cells, respectively. The morphology index (perimeter^2^/4π area) was calculated using the area and perimeter of BMDCs or FRCs by manually drawing around the cell shape using F-actin staining.

### Live imaging and analysis

Control and PDPN, CD44 and/or CD9 KO FRC cells (5×10^4^ cells per well) were seeded in 12-well plates and incubated overnight at 37°C, 10% CO_2_. The next day, cells were imaged every 10 min on a Nikon Ti inverted microscope fitted with a Nikon DS-Qi2 CMOS camera and a controlled 37°C and 5% CO_2_ atmosphere chamber. For motility analysis, cells were manually tracked using Fiji/ImageJ Manual Tracker plugin. Individual cells were tracked by clicking the centre of the nucleus from the start of a file or immediately after cell division, and until the next division, the end of the file or until the nucleus reached the boundaries of the image field. The distance travelled between frames and position per frame was calculated by Fiji. R software was used to calculate the cumulative distance travelled per cell and to plot the individual trajectories (R Core Team, https://www.R-project.org/). To show the maximum distance travelled by cells, overlaid semi-transparent circles were plotted with radii equivalent to the maximum distance travelled by each cell from the origin (calculated as 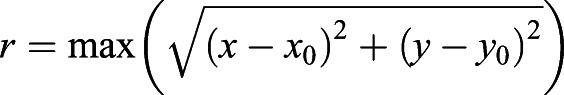.

### RNA-seq data

RNA-seq data (Martinez et al., 2019; used in Fig. 2A) are publicly available through UCL research data repository: 10.5522/04/c.4696979.

### Statistics

Statistical differences between two groups were determined using unpaired two-tailed Student's *t*-tests. Statistical differences between two different parameters were determined using one-way ANOVA with Tukey's multiple comparisons test. Statistical differences between more than two groups were determined using two-way ANOVA with Tukey's multiple comparisons test, or, in the case of non-Gaussian distribution, Kruskal–Wallis test with Dunn's multiple comparisons. Statistical tests were performed using GraphPad Prism software (version 7), and differences were considered to be statistically significant at *P*<0.05.

## Supplementary Material

Supplementary information

Reviewer comments
