# Supplementary material for: Fibroblastic reticular cell response to dendritic cells requires coordinated activity of podoplanin, CD44 and CD9
Source: J Cell Sci. 2021 Jul 22;134(14):jcs258610. doi: 10.1242/jcs.258610 (PMC8325952; doi:10.1242/jcs.258610)
Supplement: Supplementary information [file joces-134-258610-s1.pdf]

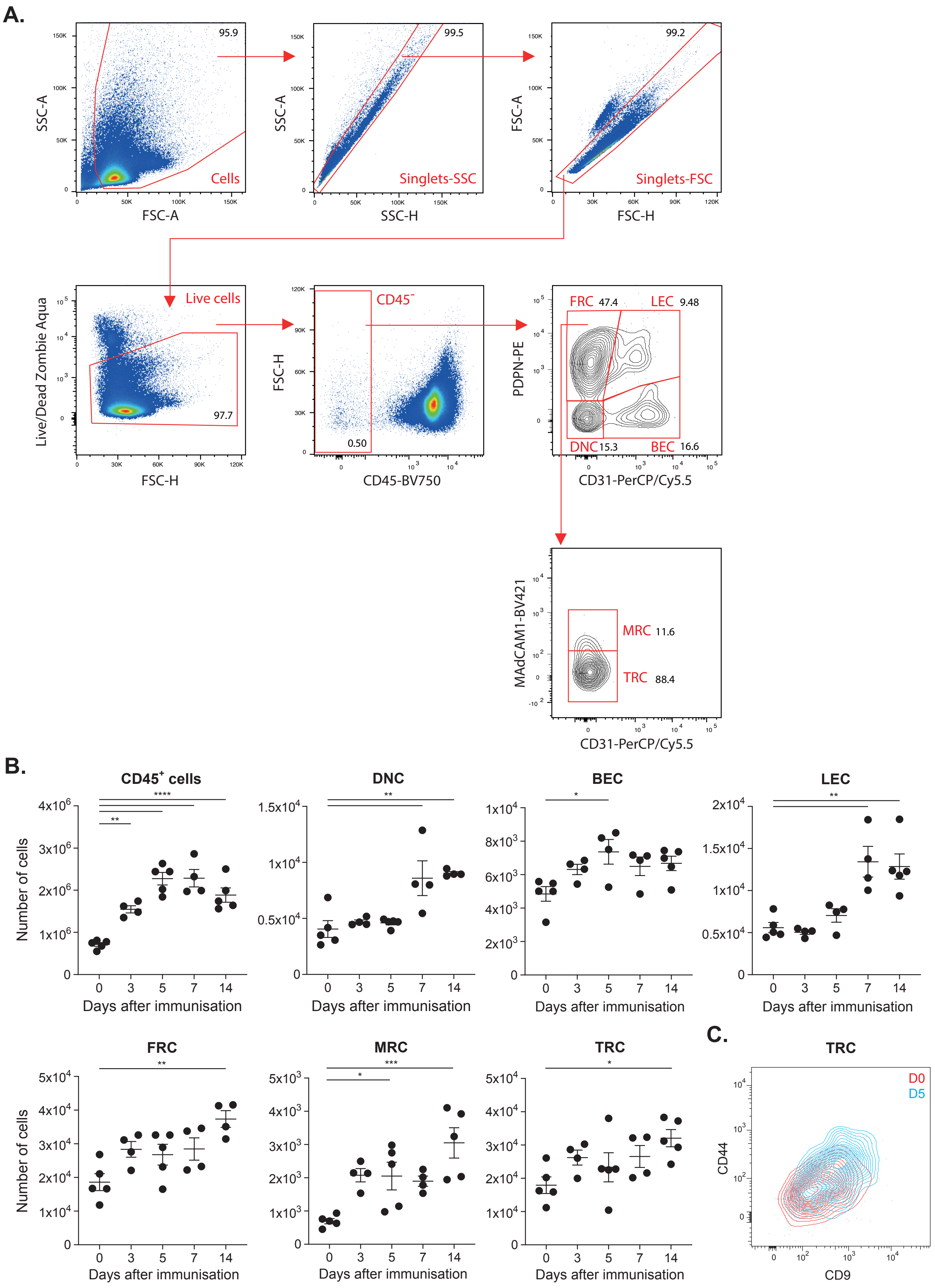

Fig. S1. A. Gating strategy for analysis of in vivo immunised lymph nodes. All gates are based on **FM**Os (fluorescence minus one samples) and relevant controls. Numbers indicate percentage of parental population. BECs = blood endothelial cells, BV = Brilliant Violet, DNCs = double negative cells, FRCs = fibroblastic reticular cells, LECs = lymphatic endothelial cells, MRCs = marginal reticular cells, TRCs = T-zone FRCs. B. Analysis by flow cytometry of number of leukocytes (CD45+) and indicated stromal cell populations in cell suspensions of inguinal lymph nodes from C57BL/6 mice immunized with IFA/OVA for indicated time points. One-way ANOVA with Tukey's multiple comparisons, *p<0.05, **p<0.01, ***p<0.001, ****p<0.0001. A-B. n=4-5 mice per time point. C. Analysis by flow cytometry of CD9 (x-axis) and CD44 (y-axis) surface protein expression on TRCs at steady-state (D0; red) and 5 days after immunization (D5; blue). One representative contour plot for each time point is shown.

Journal of Cell Science • Supplementary information

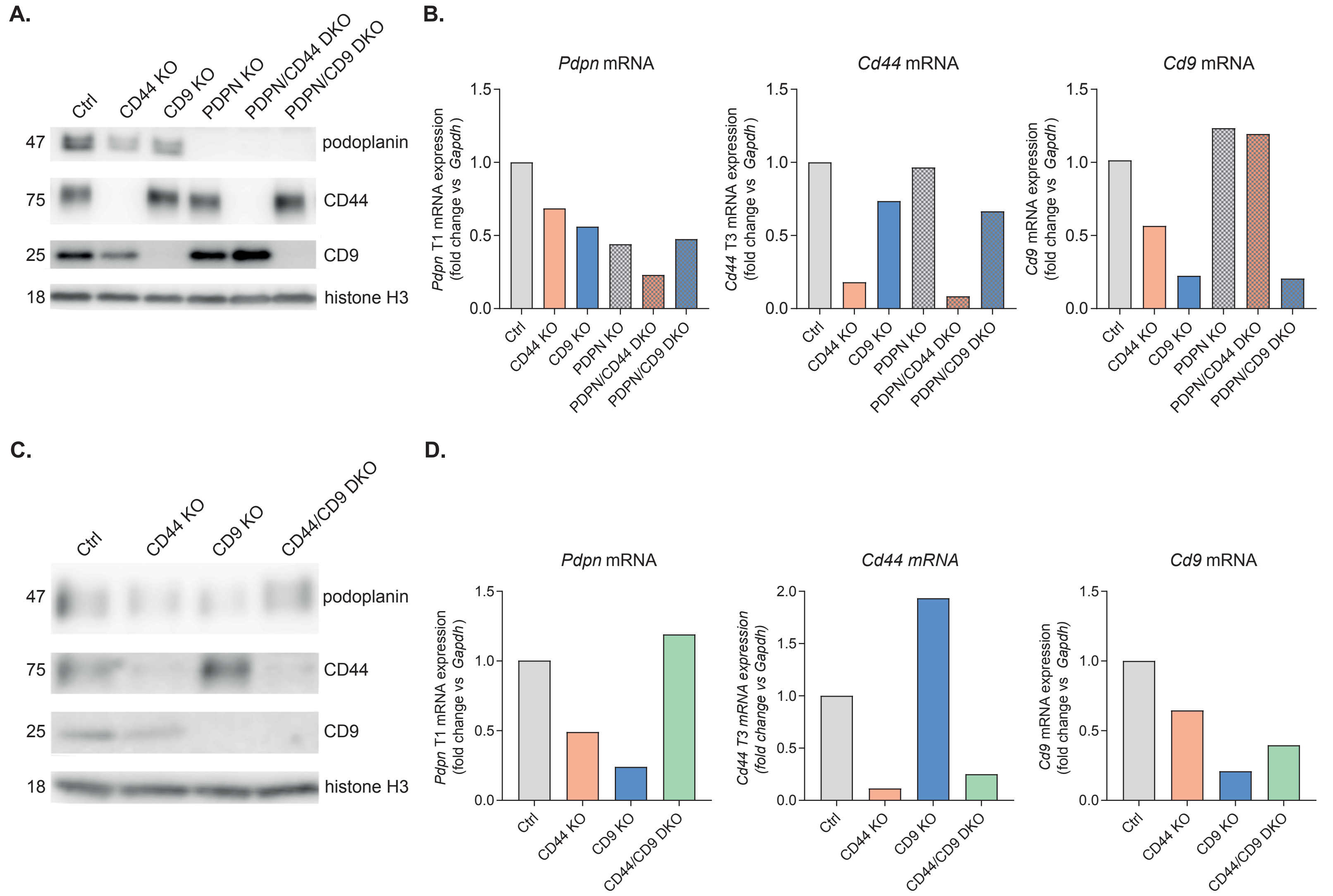

Fig. S2. Validation of podoplanin, CD44 and/or CD9 KO FRC cell lines.
A,C. Western blot analysis of total podoplanin (47kDa), CD44 (75kDa) and CD9 (25kDa) expression in Ctrl, CD44 KO, CD9 KO, PDPN KO, PDPN/CD44 DKO, PDPN/CD9 DKO (A) and CD44/CD9 DKO (C) FRC cell lines. Histone H3 (18kDa) is used as loading control. B,D. mRNA expression of PDPN transcript variant 1 (Pdpn T1; left), Cd44 (middle) and Cd9 (right) in Ctrl, CD44 KO, CD9 KO, PDPN KO, PDPN/CD44 DKO, PDPN/CD9 DKO (B) and CD44/CD9 DKO (D) FRC cell lines. Gene expression is shown as fold change of the housekeeping gene Gapdh.

Journal of Cell Science • Supplementary information

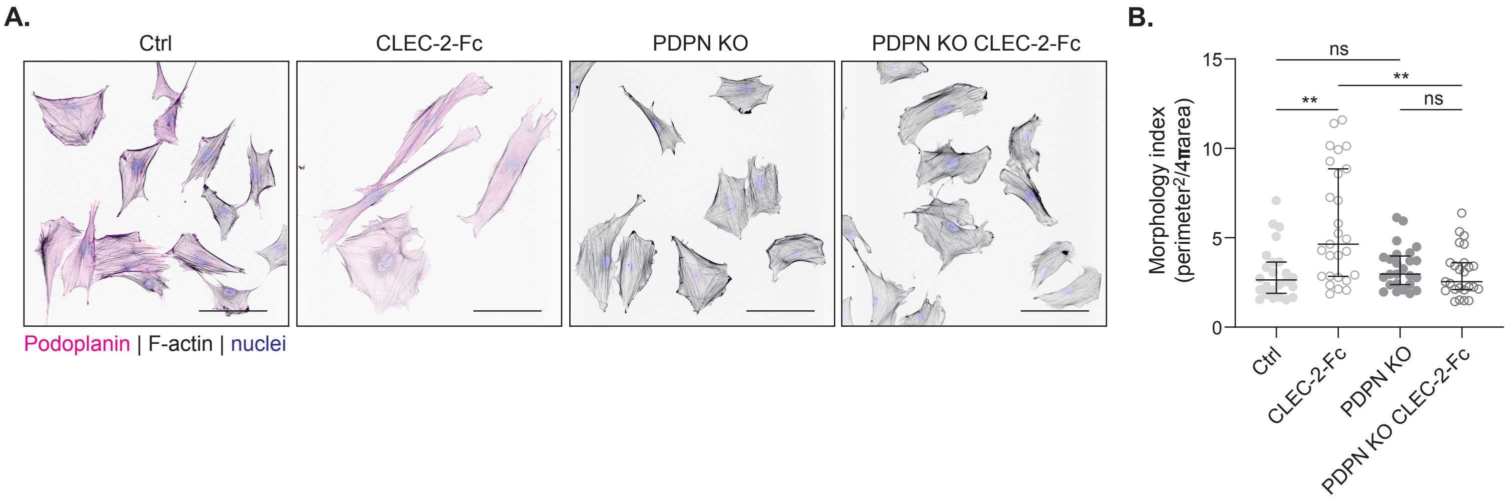

Fig. S3. CLEC-2 specifically inhibits podoplanin-driven FRC contractility.
A. Immunofluorescence of podoplanin (magenta), F-actin (black) and cell nuclei (blue) in Ctrl, CLEC-2-Fc, PDPN KO and PDPN KO CLEC-2-Fc FRC cell lines. Maximum Z stack projections of representative images are shown. The scale bars represent 100 microns. B. Morphology index (=perimeterˆ2/4*pi*area) of FRCs. Dots represent single cells (n=24-27). Error bars represent median with interquartile range. Kruskal-Wallis test with Dunn's multiple comparisons, **p<0.01. ns, not significant.
