# Supplementary material for: Fibroblastic reticular cell response to dendritic cells requires coordinated activity of podoplanin, CD44 and CD9
Source: J Cell Sci. 2021 Jul 22;134(14):jcs258610. doi: 10.1242/jcs.258610 (PMC8325952; doi:10.1242/jcs.258610)
Supplement: Reviewer comments [file joces-134-258610_review_history.pdf]

Fibroblastic reticular cell response to dendritic cells requires coordinated activity of podoplanin, CD44 and CD9

Charlotte M. de Winde, Spyridon Makris, Lindsey J. Millward, Jesús A. Cantoral-Rebordinos, Agnesska C. Benjamin, Victor G. Martinez and Sophie E. Acton
DOI: 10.1242/jcs.258610

Editor: Daniel Billadeau

Review timeline

Submission to Review Commons:	23 November 2021
Submission to Journal of Cell Science:	4 March 2021
Editorial decision:	15 March 2021
First revision received:	18 June 2021
Editorial decision:	22 June 2021

Reviewer 1

Evidence, reproducibility and clarity

So far, our understanding of podoplanin function in the biology of lymph node fibroblasts (FRC) was incomplete, and hardly anything was known on CD44 and CD9 in these cells. De Winde and colleagues provide a carefully performed study, mostly on an FRC line in vitro, of the various roles podoplanin and its partner proteins CD44 and CD9 play in FRCs in response to interaction with Clec2+ dendritic cells, with implications for FRC cytoskeleton remodeling, relaxation, protrusion and spread. They represent a logical follow-up of previous studies by this laboratory and highlight an intricate interplay between podoplanin, CD44 and CD9 acting in a two-step process of relaxation followed by cell spreading. The data were generated with state of the art tools, presented in nice figures, with the results being well described and discussed (with few exceptions listed below), and the conclusions being well supported by the data.

Major comments:
1) Figure 1: The stretching of FRC allowing the initial LN swelling is presumably most important in the very early phase before proliferation starts with the latter varying in the different immunization schemes used by various labs. Migratory Clec2+ DC arrive already on day 1 and may get to peak numbers on day 2 (Acton Nature 2014) when Clec2-mediated signals are most relevant for the processes described but data are only provided for day 3 onward. FRC size and activity (FSC and SSC) can change within hours of immunization (Yang et al., PNAS 2014) and so it would be important to show data for the relevant time window by including an earlier time point in figure 1 (day 1 and day 2, or day 1.5).

2) Figure 1: FRC grow in size after immunization (Yang et al., 2014) and so the increase in popoplanin, CD44 and CD9 is only relative; meaning the density at the surface may not be changed. The authors should provide data on the FSC changes for the time points investigated (in the supplement) and discuss briefly this point. FACS histograms should be shown for 1e-g to show the profile of expression of these molecules in homeostasis vs for example day 5 after immunization (allowing also to judge whether all cells within the subset are positive). The legend should state whether a relative MFI is shown.

3) Please add statistical significance when appropriate (for example Fig. 2c/d)

© 2021. Published by The Company of Biologists under the terms of the Creative Commons Attribution License (https://creativecommons.org/licenses/by/4.0/).

4) Regarding pdpn detection: for western blot another antibody was used than for cell surface protein expression analysis. Given that pdpn gets strongly glycosylated it is possible that the two reagents recognize different forms while the authors do not discuss it but emphasize the discrepancy between western blot results and surface expression.

5) Figure 2e indicates the distinct clustering of pdpn/CD44 vs pdpn/CD9. It seems that in both cases there is a nonrandom distribution with complexes still clustering but with different proximity. Alternatively, the CD44 staining may be more prominent than the CD9 and so there may be less white colocalization, which could point towards a difference in the CD44 cluster size or in the efficiency of antibody labeling. Can the authors discuss this point? By flow cytometry one does not get the impression of different CD44 vs CD9 expression (if the same abs were used). Would a triple staining allow to show better the different pools of podoplanin, or to highlight that CD44 is connecting pdpn with CD9 when not present at the edge of the cell?

Minor comments:
1) The representation and referencing of the current literature on LN swelling and FRC effects can be improved, as it is biased towards the previous studies by the authors. This concerns the start of the results and the discussion sections, including the concepts of FRC size increase followed by proliferation (Yang et al., PNAS 2014; cited in some spots) and the role of dendritic cells in this process (include Yang et al., PNAS 2014, Kumar et al. 2015, not cited) or network connectivity in immune response (Yang et al., PNAS 2014). The increase in podoplanin expression upon lymph node swelling (Fig. 1) has been previously reported and is therefore not that surprising; the studies of Yang et al, PNAS 2014, Acton et al. Nature 2014 and Kumar et al. Immunity 2015 should be cited in this context. Previous evidence also points towards LTbR stimulation of FRC by DCs for podoplanin expression and pdpn- mediated cell survival (Kumar et al) which could be used to enrich the discussion.

2) The FRC subsets are oversimplified relative to the current literature. If TRC are considered the remaining fraction once MRC have been gated from the FRC population, then the use of this terminology should be explained in a sentence to state that this includes another major FRC subset of the medulla (Sitnik et al. Cell reports 2016, Huang et al., PNAS 2018; Rodda et al., Immunity 2018).

3) Figure 2 legend: please state on top or in each section that these are FRC lines investigated.

4) Fig 2a/b: less surface expression can also be due to differences in translation or transport to the surface.

Significance

These novel results, together with previous in vivo results, provide a deeper understanding of the process of lymph node swelling when the fibroblast network expands, highlighting some of the cellular processes and molecular players involved, and suggests new molecules to be studied in this process in vivo. This study should be of interest to people interested in FRC and lymph node swelling, or in the molecular processes of cell polarization, spreading and migration.

My expertise as well as the one of my co-reviewer (postdoc in my lab) are in lymph node FRC in development, homeostasis and immunity.

Reviewer 2

Evidence, reproducibility and clarity

de Winde and coworkers study in this work the events triggered by the binding of CLEC-2 to podoplanin on the surface of FRC cells. In vivo, this interaction is pivotal for lymph node expansion during the adaptive immune response since CLEC-2 on dendritic cells (DCs) inhibits podoplanin-induced actomyosin hypercontractility allowing FRC spreading. By using an immortalized FRC cell line either exposed to recombinant CLEC-2 or expressing CLEC-2-Fc (in order to mimick prolongued CLEC-2 exposure from migratory DC cells arriving into the lymph node) and performing knockdown (shRNA) and KO (CRISPR/Cas9) experiments, the authors show that CLEC-2 upregulate podoplanin

© 2021. Published by The Company of Biologists under the terms of the Creative Commons Attribution License (https://creativecommons.org/licenses/by/4.0/).

and CD44 expression at the transcriptional level. Podoplanin co-localizes with CD44 and its other partner CD9 in different regions of the plasma membrane, and both partners temper podoplanin-driven contractility. Podoplanin controls FRC cell motility and polarity while CD9 and CD44 control cell-cell interactions independently of podoplanin.

This is a well-written piece of work with interesting results showing an active role of podoplanin beyond CLEC-2-podoplanin-driven relaxation of actomyosin contractility, which in collaboration with CD44 and CD9 promote FRC protrusions and spread. However, there are some concerns that limit the scope and significance of the article:

1. All novel and significant results come from in vitro experiments using only a FRC cell line immortalized by infection with HPV-E6-encoding retrovirus. It is clear that investigating the roles of podoplanin, CD44 and CD9 on FRC expansion in vivo, in the context of the adaptive immune response, is extremely difficult and challenging. But at least two immortalized FRC cell lines should have been used in order to obtain more consistent results.

2. We miss a proper characterization of knockdown and knockouts of the different molecules. These should be shown at least as supplementary data.

3. In Figure 2, where the expression of podoplanin, CD44 and CD9 are shown at the level of mRNA, protein or cell surface, only partial results are shown. Why not Western blots for CD44 and CD9 are presented in order to see changes in total protein expression?

4. It is shown in Figure 2 that PDPN knockdown reduces CD44 and CD9 mRNA and cell- surface expression. On the other hand, FRCs KO for CD44 and CD9 showed reduced cell- surface PDPN expression, revealing interdependence in the expression of these proteins. But, does silencing of CD44 and CD9 affect PDPN mRNA and protein expression? It should be important to unveil whether a reciprocal regulation in the expression of these proteins exist.

5. How co-localization is measured in Figure 2e in single sections or in the maximum Z stack projections shown? In general, the immunofluorescence analysis reported is deficient and not clear. It seems to contain a high degree of unspecificity.
Figure 2e shows co-localization of PDPN with CD44 and CD9 in FRC control and CLEC-2- Fc-expressing cells, but does CD9 co-localize with CD44? Did the authors explore the existence of a ternary complex? In this respect, authors assume that co-localization is equivalent to interaction, and this might not be true.

6. In Figure 3, contractility of CD44/CD9 double KO FRCs is highly reduced upon expressing CLEC-2, what allows the authors to conclude that hypercontractility in the double KO is podoplanin-dependent. Although podoplanin is the only natural ligand of CLEC-2, this assertion should be confirmed by silencing podoplanin expression. It should be taken into account that the double KO of CD44/CD9 reduces cell surface podoplanin expression (Fig. 2d).

7. In Figure 4a and b, the way migration parameters are presented are somehow confusing. I cannot understand what coloured circles represent in panel b.

8. In Figure 5, the authors state an increase of ARPC2 staining in membrane protrusions of FRCs lacking PDPN, CD44 or CD9, which I do not see so clearly in the pictures shown.
Presence of ARPC2 on membrane protrusions should be quantified by some mean in a significant number of cells.

9. Since CLEC-2 binding inhibits podoplanin-dependent activation of RhoA/RhoC signaling activity, which are the signaling events associated with FRC spreading and elongation? The lack of this type of studies make this paper somehow incomplete.

Minor points:

-Authors state referring the published work of another group that the interaction of podoplanin with CD44 is mediated by the extracellular domains (ref. 19), although it was recently reported that this interaction also involves the transmembrane and cytosolic regions (ref. 20). This sentence

© 2021. Published by The Company of Biologists under the terms of the Creative Commons Attribution License (https://creativecommons.org/licenses/by/4.0/).

is wrong, or at least confusing, since in the latter reference the above mentioned group retracted their former conclusion, showing that PDPN-CD44 interaction is mainly mediated by the transmembrane domains, with the cytosolic and extracellular regions modulating this interaction.

-In page 5, FRCs moving at speeds <0.2 ?/min (□m?)

Significance

This paper continues a series of studies performed by the group focused to understand the role of podoplanin in the remodelling of lymph nodes during the adaptive immune response. In FRCs, podoplanin promotes actomyosin contractility through its binding to ERM proteins and activation of RhoA/C GTPase, and binding of CLEC-2 located on migratory DCs inhibits podoplanin-induced contractility, resulting in FRC spreading and elongation to allow a rapid lymph node expansion. In a previous report, de Winde and colleagues suggested that upon binding of CLEC-2 to FRCs, podoplanin is recruited to cholesterol-rich domains (lipid rafts) where interacts with membrane partners CD44 and CD9 to form cell protrusions and to spread, issues that have studied in the present paper.
While the results obtained are relevant for those readers interested in understanding the role of podoplanin in lymph node remodelling or cell motility, this work is weakened by several facts: i) results come from only a FRC cell line; ii) no interaction of podoplanin with CD44 and CD9 is shown, but only immunofluorescence co-localization; iii) no signaling events downstream the CLEC-2-podoplanin axis associated with FRC spreading and elongation have been studied.

Reviewer 3

Evidence, reproducibility and clarity

The authors in this manuscript are investigating the mechanisms by which Clec-2/podoplanin interactions between dendritic cells (DCs) and fibroblastic reticular cells (FRCs) facilitate FRC spreading and lymph node (LN) expansion. This paper makes a significant contribution to understanding how expansion of the FRC network in LNs is accomplished during an infection by DCs. Previous studies have established the critical role of CLEC2 on DCs in LN expansion. What is not understood is how CLEC2 binding to FRCs facilitates their expansion and how podoplanin interacts with its partners in FRCs to accomplish this in response to DCs.

In the first two studies presented, the authors find that 1) podoplanin and its partner proteins CD44 and CD9 are coregulated in FRCs by Clec2, 2) podoplanin, CD44, and CD9 are differentially expressed in different LN stromal populations, and 3) CD44 and CD9 co- localize with podoplanin in different areas of the cell membrane. Moreover, they show that CD44/podoplanin complexes are induced by CLEC2, while CD9/podoplanin complexes are not. They next explored the extent to which CD44 or CD9 control different aspects of FRC function, including contractility, migration, alignment, cell-cell network interactions, and membrane protrusions. Here they find that CD44 is required for contractility, suppression of protrusions, suppression of migration, and enhances cell-cell interactions; while CD9 suppresses protrusions, migration, and cell-cell interactions, with some effects being independent of podoplanin. Neither is required for alignment. These studies nicely define different roles of podoplanin, CD44, and CD9 in FRCs.
However, there is a major concern that some of the studies appear to be have been conducted on FRC cells without any exposure to Clec2, while others included Clec2. Thus, it is hard to distinguish specifically what functions of FRC studied are simply intrinsic to the FRC independent of Clec2 versus those that are specifically controlled by Clec2. These creates some confusion in their conclusions.

Additional points to address include:

1) Concluding paragraph for Figure 2 data near the end of page 3 is not quite correct with respect to what is happening with CD9. In addition, to support their conclusion that CD9/PDPN and CD44/PDPN complexes maybe different, colocalization experiments with CD9/CD44 should be done.

2) No stats are provided for the graphs in Fig 2c and 2d.

© 2021. Published by The Company of Biologists under the terms of the Creative Commons Attribution License (https://creativecommons.org/licenses/by/4.0/).

3) In Fig. 3, Knock-down of both CD9 and CD44 together are required to reverse the contractility induced by Pdpn, while overexpression of either are sufficient to rescue contractility. Yet, only the CD44/pdpn complexes are altered upon Clec-2 binding. Thus, it is not clear how biologically important CD9 is to the process of contractility in response to Clec2? What happens to FRC expansion/function in CD9 knock out mice?

4) An important conclusion from the data in Fig. 4 is that CD44 and CD9 each independently limit baseline FRC motility, but have no impact on alignment. How do these cells respond to motility and alignment when subjected to rCLEC2? Ditto for Fig. 5, how are FRC-FRC interactions impacted by exposure to Clec2. These studies would be much stronger and more robust conclusions drawn about their role in Clec2-dependent processes if this is investigated.

5) The data in Fig. 6 is very strong, as it the most biologically relevant and really highlights the dependency on both CD9 and CD44 for the morphological response.

Significance

This paper makes a significant contribution to understanding how expansion of the FRC network in LNs is accomplished during an infection by DCs. Previous studies have established the critical role of CLEC2 on DCs in LN expansion. What is not understood is how CLEC2 binding to FRCs facilitates their expansion and how podoplanin interacts with its partners in FRCs to accomplish this in response to DCs. These studies nicely define different roles of podoplanin, CD44, and CD9 in FRCs.

Audience: Those interested in lymphocyte/immune cell biology, cell-cell signaling mechanisms, tetraspanins and associated proteins, My expertise: tetraspanins, signaling, and motility

---

Author response to reviewers' comments

We thank the reviewers for their constructive suggestions and complimentary comments on our manuscript. We are pleased that the reviewers, with expertise spanning lymphoid tissue biology to tetraspanin function, have found our work to be well conducted and of interest to a broad readership. The majority of the reviewer's comments can be addressed with changes to the text and by the addition of existing data which was not shown in the first submission. Below, we detail in our response what changes to our manuscript we propose to address each point raised.

Reviewer #1 (Evidence, reproducibility and clarity (Required)):

So far, our understanding of podoplanin function in the biology of lymph node fibroblasts (FRC) was incomplete, and hardly anything was known on CD44 and CD9 in these cells. De Winde and colleagues provide a carefully performed study, mostly on an FRC line in vitro, of the various roles podoplanin and its partner proteins CD44 and CD9 play in FRCs in response to interaction with Clec2+ dendritic cells, with implications for FRC cytoskeleton remodeling, relaxation, protrusion and spread. They represent a logical follow-up of previous studies by this laboratory and highlight an intricate interplay between podoplanin, CD44 and CD9 acting in a two-step process of relaxation followed by cell spreading. The data were generated with state of the art tools, presented in nice figures, with the results being well described and discussed (with few exceptions listed below), and the conclusions being well supported by the data.

The thank the reviewer for their positive comments and we would reiterate that our data extend our understanding of fibroblastic reticular network remodeling for lymph node expansion, beyond the PDPN/CLEC-2 signalling axis.

Major comments:

1) Figure 1: The stretching of FRC allowing the initial LN swelling is presumably most important

© 2021. Published by The Company of Biologists under the terms of the Creative Commons Attribution License (https://creativecommons.org/licenses/by/4.0/).

in the very early phase before proliferation starts with the latter varying in the different immunization schemes used by various labs. Migratory Clec2+ DC arrive already on day 1 and may get to peak numbers on day 2 (Acton Nature 2014) when Clec2-mediated signals are most relevant for the processes described but data are only provided for day 3 onward. FRC size and activity (FSC and SSC) can change within hours of immunization (Yang et al., PNAS 2014) and so it would be important to show data for the relevant time window by including an earlier time point in figure 1 (day 1 and day 2, or day 1.5).

The reviewer is correct that infiltrating DCs peak at day 2. We have therefore decided to look at day 3-5 post-immunization to look at the FRC response after DCs have migrated into the lymph node. This time frame aligns with the phase of FRC elongation and spreading before proliferation (with the particular immunization (IFA-OVA) we have used), and is documented in earlier published studies by our group and others. While it would also be interesting to study the very acute changes to FRC phenotype, the main aim of figure 1 is to compare the FRC phenotype in steady state versus stretching through acute lymph node expansion, and for this purpose day 3 and day 5 are the most relevant timepoints for IFA-OVA immunization.

2) Figure 1: FRC grow in size after immunization (Yang et al., 2014) and so the increase in popoplanin, CD44 and CD9 is only relative; meaning the density at the surface may not be changed. The authors should provide data on the FSC changes for the time points investigated (in the supplement) and discuss briefly this point. FACS histograms should be shown for 1e-g to show the profile of expression of these molecules in homeostasis vs for example day 5 after immunization (allowing also to judge whether all cells within the subset are positive). The legend should state whether a relative MFI is shown.

The reviewer correctly highlights that FRCs increase in both length and volume to facilitate acute lymph node expansion. This change in cell size then does make comparison of surface expression of markers problematic since the FRCs will have a large surface area.

To address this, we propose to include the FSC data for LECs, MRCs and TRCs, as a measure of cell size, for all timepoints in Figure 1 and discuss the data in the manuscript. Furthermore, as requested, we will provide representative FACS histograms for podoplanin, CD44 and CD9 expression in LECs, MRCs and TRCs for all time points. We propose to keep the current panels e-g in Figure 1 showing the summary data (n=5), alongside the representative histograms. The changes requested only require alternative presentation of the existing data.

Edits to manuscript: We have now added to the figure legend of Figure 1 that "relative gMFI for each marker per cell type is shown" (lines 847-848).

3) Please add statistical significance when appropriate (for example Fig. 2c/d)

We will add statistical significance to Figure 2, panels c-e and where data is not statistically significant, this will be made clear in the figure legends (n.s.).

4) Regarding pdpn detection: for western blot another antibody was used than for cell surface protein expression analysis. Given that pdpn gets strongly glycosylated it is possible that the two reagents recognize different forms while the authors do not discuss it but emphasize the discrepancy between western blot results and surface expression.

The reviewer is correct that the two different antibodies may recognize different pools of podoplanin protein and therefore cannot be directly compared. The anti-podoplanin antibody we used for flow cytometry (clone 8.1.1) does not recognize podoplanin on Western blot, for which we used antibody clone 8F11. However, we do not directly compare levels of podoplanin protein in different cellular compartments. We show only that following CLEC-2-dependent transcriptional upregulation of podoplanin that CLEC-2 stimulation results in increased podoplanin protein expression. We feel that showing both total protein level by western blotting, and surface expression by flow cytometry make this point more strongly than showing the result by one or the other method. We can edit the text to add a comment addressing this technical point.

5) Figure 2e indicates the distinct clustering of pdpn/CD44 vs pdpn/CD9. It seems that in both

© 2021. Published by The Company of Biologists under the terms of the Creative Commons Attribution License (https://creativecommons.org/licenses/by/4.0/).

cases there is a nonrandom distribution with complexes still clustering but with different proximity. Alternatively, the CD44 staining may be more prominent than the CD9 and so there may be less white colocalization, which could point towards a difference in the CD44 cluster size or in the efficiency of antibody labeling. Can the authors discuss this point? By flow cytometry one does not get the impression of different CD44 vs CD9 expression (if the same abs were used). Would a triple staining allow to show better the different pools of podoplanin, or to highlight that CD44 is connecting pdpn with CD9 when not present at the edge of the cell?

The data presented in Figure 2e show that there is some degree of colocalization of podoplanin with CD44, and with CD9, but as presented cannot fully address the reviewer's question.

We have also conducted triple staining of podoplanin, CD44 and CD9. Below, we show that CD44 and CD9 are not expressed in completely separate membrane regions, and therefore we cannot exclude the possibility that all 3 partner proteins may exist together in some areas of the membrane. We do not find any change to CD44/CD9 colocalization in the presence of CLEC-2.

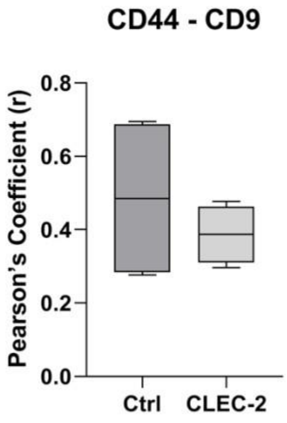

We propose to include this data in the revised manuscript, and to show the triple staining of podoplanin, CD44 and CD9 in order to add some discussion of this point to the text.

Another addition we propose to make to figure 2, or to a supplementary figure, is to include flow cytometry data as dot plots (CD44 vs. CD9 surface expression on LECs, MRCs and TRCs) to show the heterogeneity of their expression at a single cell level on these stromal cells.

Minor comments:

1. The representation and referencing of the current literature on LN swelling and FRC effects can be improved, as it is biased towards the previous studies by the authors. This concerns the start of the results and the discussion sections, including the concepts of FRC size increase followed by proliferation (Yang et al., PNAS 2014; cited in some spots) and the role of dendritic cells in this process (include Yang et al., PNAS 2014, Kumar et al. 2015, not cited) or network connectivity in immune response (Yang et al., PNAS 2014). The increase in podoplanin expression upon lymph node swelling (Fig. 1) has been previously reported and is therefore not that surprising; the studies of Yang et al, PNAS 2014, Acton et al. Nature 2014 and Kumar et al. Immunity 2015 should be cited in this context. Previous evidence also points towards LTbR stimulation of FRC by DCs for podoplanin expression and pdpn-mediated cell survival (Kumar et al) which could be used to enrich the discussion.

We have for now included (additional) citations to *Yang et al. PNAS 2014* and *Kumar et al. Immunity 2015* in the introduction, results and discussion.

Edits to manuscript: We have added the following sentences to the discussion to address the reviewer's points:

© 2021. Published by The Company of Biologists under the terms of the Creative Commons Attribution License (https://creativecommons.org/licenses/by/4.0/).

- *Lines 392-395: "Mechanisms controlling podoplanin expression in FRCs are incompletely understood. Upregulation and maintenance podoplanin expression may be mediated by LT□R stimulation by DCs (Kumar et al., 2015) and/or altered YAP/TAZ signalling (Choi et al., 2020)."*
- *Lines 447-449: "For example, podoplanin expression on FRCs is altered in lymph node- originated haematological malignancies ((Pandey et al., 2017; Apollonio et al., 2018) and own unpublished data)."*

2. The FRC subsets are oversimplified relative to the current literature. If TRC are considered the remaining fraction once MRC have been gated from the FRC population, then the use of this terminology should be explained in a sentence to state that this includes another major FRC subset of the medulla (Sitnik et al. Cell reports 2016, Huang et al., PNAS 2018; Rodda et al., Immunity 2018).

The reviewer correctly identifies that we have grouped together all FRC subsets residing in the T cell area and labelled them as TRCs (T-zone reticular cells). We have edited the text to make this clear to a broader readership not familiar with all the identified FRC subsets.

Edits to manuscript: We have added the following sentence to the results (lines 140-142) to address this point: "We here define TRCs to include all CD31-PDPN+MAdCAM-1- FRC subsets (Sitnik et al., 2016; Rodda et al., 2018; Huang et al., 2019).", citing the three references above.

3. Figure 2 legend: please state on top or in each section that these are FRC lines investigated.

We have now included throughout the results of Figures 2-6 and in the corresponding figure legends that FRC cell lines have been investigated. The original immortalised FRC cell lines are characterised in the publication where they are first used (Acton *et al.* Nature 2014), and all knock out lines are generated from this polyclonal cell line.

4. Fig 2a/b: less surface expression can also be due to differences in translation or transport to the surface.

We thank the reviewer for bringing up this point. We have now included these options in the main text. We agree that changes to podoplanin surface expression can be due to changes to translation and/or transport, in addition to the CLEC-2-dependent transcriptional increase of podoplanin expression.

Edits to manuscript: (line 188-189), together with the options we already mentioned (changes in protein degradation or transcription): "Using our in vitro model system, we show that CLEC-2 binding is sufficient to increase podoplanin protein expression in an immortalized FRC cell line (Acton et al., 2014; Martinez et al., 2019) (Fig. 2a), suggesting that regulation of podoplanin by CLEC-2 occurs either by inhibiting protein degradation, increased transport to the cell surface, differences in translation or changes at the transcriptional level."

Reviewer #1 (Significance (Required)):

These novel results, together with previous in vivo results, provide a deeper understanding of the process of lymph node swelling when the fibroblast network expands, highlighting some of the cellular processes and molecular players involved, and suggests new molecules to be studied in this process in vivo. This study should be of interest to people interested in FRC and lymph node swelling, or in the molecular processes of cell polarization, spreading and migration.

My expertise as well as the one of my co-reviewer (postdoc in my lab) are in lymph node FRC in development, homeostasis and immunity.

Reviewer #2 (Evidence, reproducibility and clarity (Required)):

de Winde and coworkers study in this work the events triggered by the binding of CLEC-2 to

© 2021. Published by The Company of Biologists under the terms of the Creative Commons Attribution License (https://creativecommons.org/licenses/by/4.0/).

podoplanin on the surface of FRC cells. In vivo, this interaction is pivotal for lymph node expansion during the adaptive immune response since CLEC-2 on dendritic cells (DCs) inhibits podoplanin- induced actomyosin hypercontractility allowing FRC spreading. By using an immortalized FRC cell line either exposed to recombinant CLEC-2 or expressing CLEC-2-Fc (in order to mimick prolongued CLEC- 2 exposure from migratory DC cells arriving into the lymph node) and performing knockdown (shRNA) and KO (CRISPR/Cas9) experiments, the authors show that CLEC-2 upregulate podoplanin and CD44 expression at the transcriptional level. Podoplanin co-localizes with CD44 and its other partner CD9 in different regions of the plasma membrane, and both partners temper podoplanin- driven contractility. Podoplanin controls FRC cell motility and polarity while CD9 and CD44 control cell-cell interactions independently of podoplanin.

This is a well-written piece of work with interesting results showing an active role of podoplanin beyond CLEC-2-podoplanin-driven relaxation of actomyosin contractility, which in collaboration with CD44 and CD9 promote FRC protrusions and spread. However, there are some concerns that limit the scope and significance of the article:

We thank the reviewer for their comments which reflect a significant interest in podoplanin signaling in FRCs and other contexts.

1. All novel and significant results come from in vitro experiments using only a FRC cell line immortalized by infection with HPV-E6-encoding retrovirus. It is clear that investigating the roles of podoplanin, CD44 and CD9 on FRC expansion in vivo, in the context of the adaptive immune response, is extremely difficult and challenging. But at least two immortalized FRC cell lines should have been used in order to obtain more consistent results.

FRCs have been notorious difficult to immortalize, and so there are not a range of FRC cell lines to use in parallel. Earlier FRC studies had been limited to using either primary cells, which are not amenable to genetic modification, or *in vivo* observations until this cell line was generated. The FRC cell line (Acton *et al.* Nature 2014) used in this study has been generated by pooling FRCs ($CD45^-$, $CD31^-$, $PDPN^+$) from lymph nodes from multiple mice, and is maintained as a polyclonal cell line.
Furthermore, the CLEC-2-expressing, PDPN KD, PDPN KO, CD44 KO, CD9KO, CD44/CD9 DKO FRC cell lines used in our work are also all polyclonal cell lines. While we understand the point of the reviewer, in the case of this particular cell type, the models for *in vitro* experiments are very limited. We have here shown relevance of the function and coordinated expression of podoplanin, CD44 and CD9 in FRC biology in multiple ways, which we believe convincingly supports our hypothesis. We agree that this limits the study to some degree, but the next steps to validate our findings would be to generate combinations of conditional deletions in mouse models which are not available at the current time.

2. We miss a proper characterization of knockdown and knockouts of the different molecules. These should be shown at least as supplementary data.

We agree with the reviewer that it would benefit the manuscript to include more details validating the newly-generated knockout FRC cell lines used for this study. We did not notice any obvious changes in viability and proliferation rate of KO cell lines used in this study. We have confirmed loss of podoplanin, CD44 and/or CD9 surface expression by flow cytometry as shown in Fig. 2d, however western blot and qPCR data are currently not included in the manuscript. PDPN KD cell line is generated and characterized previously (Acton *et al*. Nature 2014), which is referenced in the Methods, and we have now also included this reference in the results (line 194).

We propose to make a supplementary figure including qPCR and Western blot data of podoplanin, CD44 and CD9 expression in the KO cell lines used in this study. We present these data below for the benefit of the reviewers and the editor. The double knockout of CD44 and CD9 (panels c,d - below) was generated at a later date that the single knockout cell lines (panels a,b - below)

© 2021. Published by The Company of Biologists under the terms of the Creative Commons Attribution License (https://creativecommons.org/licenses/by/4.0/).

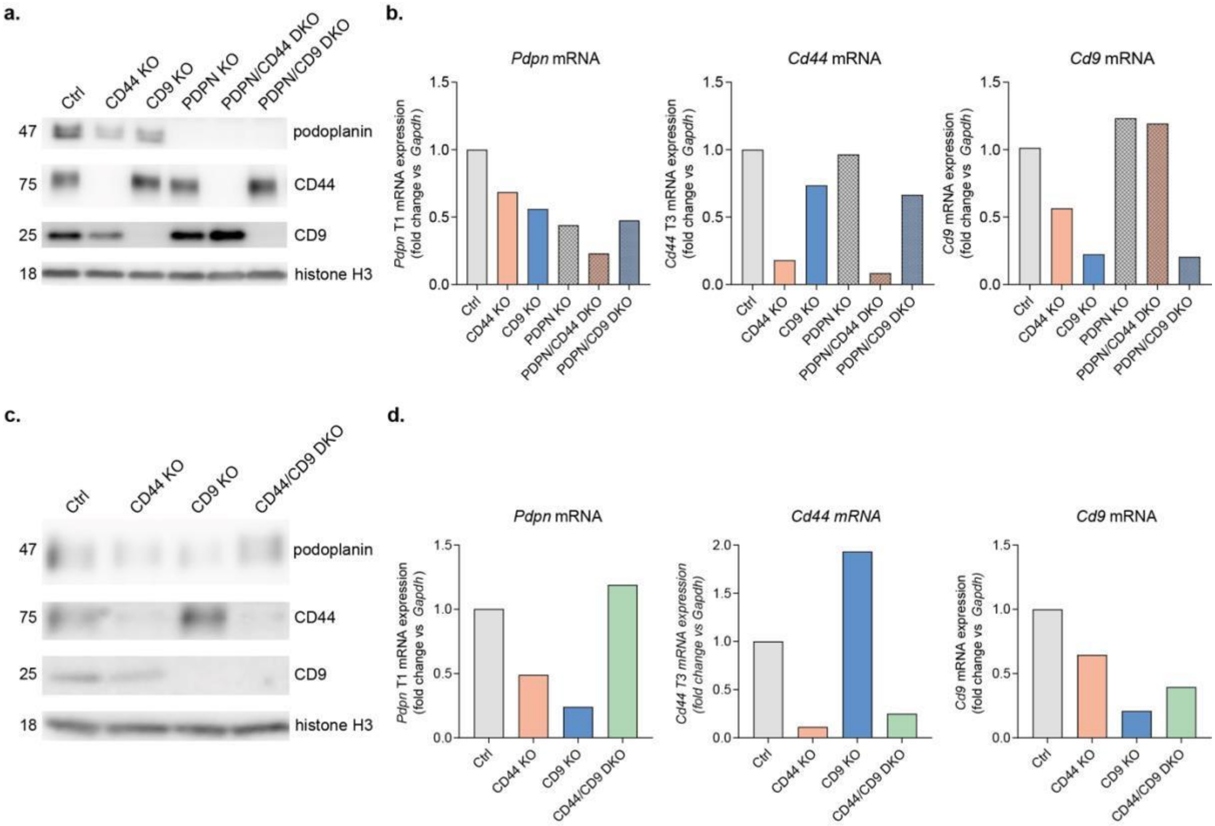

__Figure for reviewer__. Validation of podoplanin, CD44 and/or CD9 KO FRC cell lines by Western blot (a & c) and qPCR (b and d).

3. In Figure 2, where the expression of podoplanin, CD44 and CD9 are shown at the level of mRNA, protein or cell surface, only partial results are shown. Why not Western blots for CD44 and CD9 are presented in order to see changes in total protein expression?

We agree with the reviewer that the co-regulation of podoplanin, CD44 and CD9 is an interesting finding in our manuscript and should be shown in greater detail by including analysis of total protein expression in addition to the mRNA data shown. We propose to repeat these Western blots to analyze total protein expression of CD44 and CD9 in Ctrl vs. CLEC-2-Fc FRCs, and add this data to our manuscript.

4. It is shown in Figure 2 that PDPN knockdown reduces CD44 and CD9 mRNA and cell-surface expression. On the other hand, FRCs KO for CD44 and CD9 showed reduced cell-surface PDPN expression, revealing interdependence in the expression of these proteins. But, does silencing of CD44 and CD9 affect PDPN mRNA and protein expression? It should be important to unveil whether a reciprocal regulation in the expression of these proteins exist.

We agree, and as detailed above with additional data, we propose to add a supplementary figure including qPCR and Western blot data of podoplanin mRNA and protein expression in the KO cell lines used in this study (as well as CD44 and CD9 expression; see comment 2 also from reviewer 2 above).

5. How co-localization is measured in Figure 2e in single sections or in the maximum Z stack projections shown? In general, the immunofluorescence analysis reported is deficient and not clear.

It seems to contain a high degree of unspecificity.
Figure 2e shows co-localization of PDPN with CD44 and CD9 in FRC control and CLEC-2-Fc-expressing cells, but does CD9 co-localize with CD44? Did the authors explore the existence of a ternary complex? In this respect, authors assume that co-localization is equivalent to interaction, and this might not be true.

© 2021. Published by The Company of Biologists under the terms of the Creative Commons Attribution License (https://creativecommons.org/licenses/by/4.0/).

We apologies for this omission, the data in Figure 2e represents Pearson's coefficient analyzed in single sections using JaCoP plugin in Fiji/ImageJ. We have now included more detailed information in the Methods and corresponding Figure legend.

Regarding the second point exploring the possibility of a ternary complex, a question also raised by reviewer 1, we now propose to include analysis of colocalization of CD44 and CD9 to extend this figure, and to present examples of triple stained cells in steady state and in the presence of CLEC-2. Please see details above.

The reviewer is also correct that we cannot assume that colocalization is equivalent to interaction, and since we show confocal images, the resolution is not sufficient to show complexes. However, our intention with these data is to show firstly that CD9 and CD44 both colocalize with podoplanin in different regions of the cell membrane, and secondly to ask whether colocalization is altered in the presence of CLEC-2. We will edit our description of these data in addition to adding the triple staining, and ensure we do not imply that colocalization is equivalent to molecular interaction/binding.

6. In Figure 3, contractility of CD44/CD9 double KO FRCs is highly reduced upon expressing CLEC-2, what allows the authors to conclude that hypercontractility in the double KO is podoplanin-dependent. Although podoplanin is the only natural ligand of CLEC-2, this assertion should be confirmed by silencing podoplanin expression. It should be taken into account that the double KO of CD44/CD9 reduces cell surface podoplanin expression (Fig. 2d).

Although we have not generated a triple knockout cell line, we can address this important point in two ways. Firstly, it is previously published that CLEC-2 deleted dendritic cells are unable to induce FRC spreading (de Winde *et al.* J Cell Sci 2018), and in the current manuscript (Figure 6a-b) we can highlight to the reader how dendritic cell morphology does not change in co-culture with podoplanin-deficient FRCs. Secondly, we propose to show additional data comparing the morphology of control and podoplanin KO FRCs expressing CLEC-2. We have observed that CLEC-2 has no effect on FRC morphology in the absence of podoplanin.

7. In Figure 4a and b, the way migration parameters are presented are somehow confusing. I cannot understand what coloured circles represent in panel b.

We have edited the figure legend to make this representation more clear to the reader.

Edits to the manuscript: We have changed the figure legend to clarify what the coloured circles represent, which now reads (lines 901-903): "The intensity of the coloured circles represents the number of cells moving the same distance from the starting position."

We have also added an additional explanation to the Methods section (lines 618-621): "To show the maximum distance travelled by cells, overlaid semitransparent circles were plotted with radii equivalent to the maximum distance travelled by each cell from the origin (calculated as $r = \max\left(\sqrt{(x-x_0)^{2}+(y-y_0)^{2}} \right)$*)."*

8. In Figure 5, the authors state an increase of ARPC2 staining in membrane protrusions of FRCs lacking PDPN, CD44 or CD9, which I do not see so clearly in the pictures shown. Presence of ARPC2 on membrane protrusions should be quantified by some mean in a significant number of cells.

Quantification of these data would strengthen this figure. We propose to quantify ARPC2 expression on membrane protrusions by measuring mean fluorescence intensity on the cell membrane of the FRC cell lines shown in Figure 5.

9. Since CLEC-2 binding inhibits podoplanin-dependent activation of RhoA/RhoC signaling activity, which are the signaling events associated with FRC spreading and elongation? The lack of this type of studies make this paper somehow incomplete.

© 2021. Published by The Company of Biologists under the terms of the Creative Commons Attribution License (https://creativecommons.org/licenses/by/4.0/).

This is a very broad question, and reflects the fact that signaling downstream of podoplanin is still not well understood. Our data present additional roles of molecular players in addition to podoplanin, but we have not overstated our findings, and simply show that inhibition of RhoA/C is just one part of the complex response of FRCs to dendritic cells. The signaling events associated with FRC spreading include activation of Rac1 (Acton *et al*. Nature 2014), but now requires further investigation in the context of CD44 and CD9.

Minor points:

-Authors state referring the published work of another group that the interaction of podoplanin with CD44 is mediated by the extracellular domains (ref. 19), although it was recently reported that this interaction also involves the transmembrane and cytosolic regions (ref. 20). This sentence is wrong, or at least confusing, since in the latter reference the above mentioned group retracted their former conclusion, showing that PDPN-CD44 interaction is mainly mediated by the transmembrane domains, with the cytosolic and extracellular regions modulating this interaction.

We apologize for this incorrect citation, and have now changed this information in our manuscript.

Edits to manuscript: (lines 96-98): "Podoplanin interacts with CD44 through their transmembrane domains, and this interaction is modulated by both the cytosolic and transmembrane regions (Montero-Montero et al., 2020)." as well as in lines 211-212.

-In page 5, FRCs moving at speeds <0.2 ?/min (□m?)

Sorry for the confusion, this is a typo. It is indeed □m, and we have now changed this is the text (line 281).

Reviewer #2 (Significance (Required)):

This paper continues a series of studies performed by the group focused to understand the role of podoplanin in the remodelling of lymph nodes during the adaptive immune response. In FRCs, podoplanin promotes actomyosin contractility through its binding to ERM proteins and activation of RhoA/C GTPase, and binding of CLEC-2 located on migratory DCs inhibits podoplanin-induced contractility, resulting in FRC spreading and elongation to allow a rapid lymph node expansion. In a previous report, de Winde and colleagues suggested that upon binding of CLEC-2 to FRCs, podoplanin is recruited to cholesterol-rich domains (lipid rafts) where interacts with membrane partners CD44 and CD9 to form cell protrusions and to spread, issues that have studied in the present paper.
While the results obtained are relevant for those readers interested in understanding the role of podoplanin in lymph node remodelling or cell motility, this work is weakened by several facts: i) results come from only a FRC cell line; ii) no interaction of podoplanin with CD44 and CD9 is shown, but only immunofluorescence co-localization; iii) no signaling events downstream the CLEC-2- podoplanin axis associated with FRC spreading and elongation have been studied.

We thank the reviewer for their constructive comments. We believe we can address the specific questions raised as detailed above.

Reviewer #3 (Evidence, reproducibility and clarity (Required)):

The authors in this manuscript are investigating the mechanisms by which Clec-2/podoplanin interactions between dendritic cells (DCs) and fibroblastic reticular cells (FRCs) facilitate FRC spreading and lymph node (LN) expansion. This paper makes a significant contribution to understanding how expansion of the FRC network in LNs is accomplished during an infection by DCs. Previous studies have established the critical role of CLEC2 on DCs in LN expansion. What is not understood is how CLEC2 binding to FRCs facilitates their expansion and how podoplanin interacts with its partners in FRCs to accomplish this in response to DCs.

In the first two studies presented, the authors find that 1) podoplanin and its partner proteins CD44 and CD9 are coregulated in FRCs by Clec2, 2) podoplanin, CD44, and CD9 are differentially expressed in different LN stromal populations, and 3) CD44 and CD9 co-localize with podoplanin in

© 2021. Published by The Company of Biologists under the terms of the Creative Commons Attribution License (https://creativecommons.org/licenses/by/4.0/).

different areas of the cell membrane. Moreover, they show that CD44/podoplanin complexes are induced by CLEC2, while CD9/podoplanin complexes are not. They next explored the extent to which CD44 or CD9 control different aspects of FRC function, including contractility, migration, alignment, cell-cell network interactions, and membrane protrusions. Here they find that CD44 is required for contractility, suppression of protrusions, suppression of migration, and enhances cell-cell interactions; while CD9 suppresses protrusions, migration, and cell-cell interactions, with some effects being independent of podoplanin. Neither is required for alignment. These studies nicely define different roles of podoplanin, CD44, and CD9 in FRCs.

However, there is a major concern that some of the studies appear to be have been conducted on FRC cells without any exposure to Clec2, while others included Clec2. Thus, it is hard to distinguish specifically what functions of FRC studied are simply intrinsic to the FRC independent of Clec2 versus those that are specifically controlled by Clec2. These creates some confusion in their conclusions.

We thank the reviewer for their positive review. We agree that where some data are limited to the steady state functions of podoplanin, CD44 and CD9 in FRCs, the manuscript would be strengthened by comparing these phenotypes in the presence of CLEC-2 (in particular in figure 4 and 5). The additional experiments we propose to include are detailed below.

Additional points to address include:

1) Concluding paragraph for Figure 2 data near the end of page 3 is not quite correct with respect to what is happening with CD9. In addition, to support their conclusion that CD9/PDPN and CD44/PDPN complexes maybe different, colocalization experiments with CD9/CD44 should be done.

As also raised by reviewers 1 and 2, we will address this by including triple staining of podoplanin, CD44 and CD9 in steady state and in the presence of CLEC-2. We have also show above the colocalization of CD44 and CD9 which will be added to figure 2.

2) No stats are provided for the graphs in Fig 2c and 2d.

We will add statistical significance to Figure 2, panels c-e where appropriate.

3) In Fig. 3, Knock-down of both CD9 and CD44 together are required to reverse the contractility induced by Pdpn, while overexpression of either are sufficient to rescue contractility. Yet, only the CD44/pdpn complexes are altered upon Clec-2 binding. Thus, it is not clear how biologically important CD9 is to the process of contractility in response to Clec2? What happens to FRC expansion/function in CD9 knock out mice?

The reviewer raises a valid point which we do not fully address with the data as presented. It is possible that over-expression of CD9 rescues hypercontractility because the levels of expression are higher than the endogenous CD9. Since these data are ambiguous, we will edit the text to reflect the still open question of the role of CD9 in FRC contractility.

We also propose to add to figure 1 greater detail of the flow cytometry showing levels of CD9 and CD44 in stromal cell subsets, these data may allow us to comment further on this question.

The reviewer is correct, that to understand the role of CD9 in FRC function fully, we would need to look *in vivo*. However, CD9 is broadly expressed, including on other cells in the lymph node. Full CD9 KO mice are infertile (due to the pivotal role of CD9 in sperm-egg fusion; Kaji *et al*. Nat Gen 2000), which creates an extra hurdle to make conditional CD9 KO mice. Indeed, CD9 expression on T cells is required for efficient co-stimulation (Tai *et al*. J Exp Med 1996), meaning that antigen-presentation and T cell proliferation would be attenuated in CD9 KO mice, and therefore lymph nodes would not expand to the same degree as controls. As such, to investigate the *in vivo* role of CD9 on FRC expansion/function, CD9 needs to be conditionally knocked-out on FRCs, which is not currently possible.

© 2021. Published by The Company of Biologists under the terms of the Creative Commons Attribution License (https://creativecommons.org/licenses/by/4.0/).

4) An important conclusion from the data in Fig. 4 is that CD44 and CD9 each independently limit baseline FRC motility, but have no impact on alignment. How do these cells respond to motility and alignment when subjected to rCLEC2? Ditto for Fig. 5, how are FRC-FRC interactions impacted by exposure to Clec2. These studies would be much stronger and more robust conclusions drawn about their role in Clec2-dependent processes if this is investigated.

We agree that the manuscript would be strengthened if we include both steady state and CLEC-2 stimulation in figures 4 and 5. While these figures currently demonstrate important roles for podoplanin, CD44 and CD9 in steady state FRC functions, the manuscript will be more complete with these additional experiments.

We propose to include analysis of CLEC-2-expressing FRC cell lines missing podoplanin, CD44 and/or CD9 expression to Figures 4 and 5. And to quantify how alignment and FRC-FRC interactions require podoplanin, CD44 and CD9 in the presence of CLEC-2.

5) The data in Fig. 6 is very strong, as it the most biologically relevant and really highlights the dependency on both CD9 and CD44 for the morphological response.

We thank the reviewer for this positive assessment. These data represent our strongest evidence that FRCs require CD44 and CD9 in addition to podoplanin to respond to dendritic cells.

Reviewer #3 (Significance (Required)):

This paper makes a significant contribution to understanding how expansion of the FRC network in LNs is accomplished during an infection by DCs. Previous studies have established the critical role of CLEC2 on DCs in LN expansion. What is not understood is how CLEC2 binding to FRCs facilitates their expansion and how podoplanin interacts with its partners in FRCs to accomplish this in response to DCs. These studies nicely define different roles of podoplanin, CD44, and CD9 in FRCs.

Audience: Those interested in lymphocyte/immune cell biology, cell-cell signaling mechanisms, tetraspanins and associated proteins,

My expertise: tetraspanins, signaling, and motility

First decision letter

MS ID#: JOCES/2021/258610

MS TITLE: Fibroblastic Reticular Cell Response to Dendritic Cells Requires Coordinated Activity of Podoplanin, CD44 and CD9

AUTHORS: Charlotte M de Winde, Spyridon Makris, Lindsey Millward, Jesús Cantoral, Agnesska C Benjamin, Victor G Martinez, and Sophie Acton
ARTICLE TYPE: Research Article

I am sorry that has taken this long to get back to you, but there was an issue on my end. Could you please revise your manuscript as you have outlined in the cover letter. I look forward to seeing the revised version of the manuscript.

To see the reviewers' reports and a copy of this decision letter, please go to: https://submit-jcs.biologists.org and click on the 'Manuscripts with Decisions' queue in the Author Area. (Corresponding author only has access to reviews.)

We are aware that you may be experiencing disruption to the normal running of your lab that makes experimental revisions challenging. If it would be helpful, we encourage you to contact us

© 2021. Published by The Company of Biologists under the terms of the Creative Commons Attribution License (https://creativecommons.org/licenses/by/4.0/).

to discuss your revision in greater detail. Please send us a point-by-point response indicating where you are able to address concerns raised (either experimentally or by changes to the text) and where you will not be able to do so within the normal timeframe of a revision. We will then provide further guidance. Please also note that we are happy to extend revision timeframes as necessary.

Please ensure that you clearly highlight all changes made in the revised manuscript. Please avoid using 'Tracked changes' in Word files as these are lost in PDF conversion.

I should be grateful if you would also provide a point-by-point response detailing how you have dealt with the points raised by the reviewers in the 'Response to Reviewers' box. Please attend to all of the reviewers' comments. If you do not agree with any of their criticisms or suggestions please explain clearly why this is so.

First revision

Author response to reviewers' comments

We thank the reviewers for their constructive suggestions and complimentary comments on our manuscript. We are pleased that the reviewers, with expertise spanning lymphoid tissue biology to tetraspanin function, have found our work to be well conducted and of interest to a broad readership. We have now addressed the reviewer's comments with changes to the text and by the addition of new data. Below, we detail in our response what changes to our manuscript we have performed.

Reviewer #1 (Evidence, reproducibility and clarity (Required)):

So far, our understanding of podoplanin function in the biology of lymph node fibroblasts (FRC) was incomplete, and hardly anything was known on CD44 and CD9 in these cells. De Winde and colleagues provide a carefully performed study, mostly on an FRC line in vitro, of the various roles podoplanin and its partner proteins CD44 and CD9 play in FRCs in response to interaction with Clec2+ dendritic cells, with implications for FRC cytoskeleton remodeling, relaxation, protrusion and spread. They represent a logical follow-up of previous studies by this laboratory and highlight an intricate interplay between podoplanin, CD44 and CD9 acting in a two-step process of relaxation followed by cell spreading. The data were generated with state of the art tools, presented in nice figures, with the results being well described and discussed (with few exceptions listed below), and the conclusions being well supported by the data.

The thank the reviewer for their positive comments and we would reiterate that our data extend our understanding of fibroblastic reticular network remodeling for lymph node expansion, beyond the PDPN/CLEC-2 signalling axis.

Major comments:

1) Figure 1: The stretching of FRC allowing the initial LN swelling is presumably most important in the very early phase before proliferation starts with the latter varying in the different immunization schemes used by various labs. Migratory Clec2+ DC arrive already on day 1 and may get to peak numbers on day 2 (Acton Nature 2014) when Clec2-mediated signals are most relevant for the processes described but data are only provided for day 3 onward. FRC size and activity (FSC and SSC) can change within hours of immunization (Yang et al., PNAS 2014) and so it would be important to show data for the relevant time window by including an earlier time point in figure 1 (day 1 and day 2, or day 1.5).

The reviewer is correct that infiltrating DCs peak at day 2. We have therefore decided to look at day 3-5 post-immunization to look at the FRC response <u>after</u> DCs have migrated into the lymph node.

© 2021. Published by The Company of Biologists under the terms of the Creative Commons Attribution License (https://creativecommons.org/licenses/by/4.0/).

This time frame aligns with the phase of FRC elongation and spreading before proliferation (with the particular immunization (IFA-OVA) we have used), and is documented in earlier published studies by our group and others. While it would also be interesting to study the very acute changes to FRC phenotype, the main aim of figure 1 is to compare the FRC phenotype in steady state versus stretching through acute lymph node expansion, and for this purpose day 3 and day 5 are the most relevant timepoints for IFA-OVA immunization.

2) Figure 1: FRC grow in size after immunization (Yang et al., 2014) and so the increase in popoplanin, CD44 and CD9 is only relative; meaning the density at the surface may not be changed. The authors should provide data on the FSC changes for the time points investigated (in the supplement) and discuss briefly this point. FACS histograms should be shown for 1e-g to show the profile of expression of these molecules in homeostasis vs for example day 5 after immunization (allowing also to judge whether all cells within the subset are positive). The legend should state whether a relative MFI is shown.

The reviewer correctly highlights that FRCs increase in both length and volume to facilitate acute lymph node expansion. This change in cell size then does make comparison of surface expression of markers problematic since the FRCs will have a large surface area.

We have now included the FSC data for LECs, MRCs and TRCs, as a measure of cell size, for all timepoints in Figure 1 (panel e-g) and discuss the data in the manuscript (lines 150-155).

Furthermore, we have included representative FACS histograms for podoplanin, CD44 and CD9 expression in LECs, MRCs and TRCs for all time points in Figure 1, panel e-g.

We have now added to the figure legend of Figure 1 that "*relative gMFI for each marker per cell type is shown*" (lines 856-857).

3) Please add statistical significance when appropriate (for example Fig. 2c/d)

We have added statistical significance or when non-significant indicated this with "ns" to all figure panels when appropriate.

4) Regarding pdpn detection: for western blot another antibody was used than for cell surface protein expression analysis. Given that pdpn gets strongly glycosylated it is possible that the two reagents recognize different forms while the authors do not discuss it but emphasize the discrepancy between western blot results and surface expression.

The reviewer is correct that the two different antibodies may recognize different pools of podoplanin protein and therefore cannot be directly compared. The anti-podoplanin antibody we used for flow cytometry (clone 8.1.1) does not recognize podoplanin on Western blot, for which we used antibody clone 8F11. However, we do not directly compare levels of podoplanin protein in different cellular compartments. We show only that following CLEC-2-dependent transcriptional upregulation of podoplanin that CLEC-2 stimulation results in increased podoplanin protein expression. We feel that showing both total protein level by western blotting, and surface expression by flow cytometry make this point more strongly than showing the result by one or the other method. We have added a comment addressing the use of a different antibody for flow cytometry compared to Western blot (lines 187-188).

5) Figure 2e indicates the distinct clustering of pdpn/CD44 vs pdpn/CD9. It seems that in both cases there is a nonrandom distribution with complexes still clustering but with different proximity.
Alternatively, the CD44 staining may be more prominent than the CD9 and so there may be less white colocalization, which could point towards a difference in the CD44 cluster size or in the efficiency of antibody labeling. Can the authors discuss this point? By flow cytometry one does not get the impression of different CD44 vs CD9 expression (if the same abs were used). Would a triple staining allow to show better the different pools of podoplanin, or to highlight that CD44 is connecting pdpn with CD9 when not present at the edge of the cell?

The data presented in Figure 2e show that there is some degree of colocalization of podoplanin

© 2021. Published by The Company of Biologists under the terms of the Creative Commons Attribution License (https://creativecommons.org/licenses/by/4.0/).

with CD44, and with CD9, but as presented cannot fully address the reviewer's question.

We have now included data on a triple staining of podoplanin, CD44 and CD9 in Figure 2, panel f. We show that CD44 and CD9 are not expressed in completely separate membrane regions, and therefore we cannot exclude the possibility that all 3 partner proteins may exist together in some areas of the membrane. We do not find any change to CD44/CD9 colocalization in the presence of CLEC-2. We discuss this data in the text, lines 215-220.

In addition, we have now included flow cytometry data of CD44 vs. CD9 surface expression in TRCs as contour plots in Figure S1, panel c. This data shows the heterogeneity of their expression at a single cell level on these stromal cells as discussed in the main text (lines 220-223).

Minor comments:

1. The representation and referencing of the current literature on LN swelling and FRC effects can be improved, as it is biased towards the previous studies by the authors. This concerns the start of the results and the discussion sections, including the concepts of FRC size increase followed by proliferation (Yang et al., PNAS 2014; cited in some spots) and the role of dendritic cells in this process (include Yang et al., PNAS 2014, Kumar et al. 2015, not cited) or network connectivity in immune response (Yang et al., PNAS 2014). The increase in podoplanin expression upon lymph node swelling (Fig. 1) has been previously reported and is therefore not that surprising; the studies of Yang et al, PNAS 2014, Acton et al. Nature 2014 and Kumar et al. Immunity 2015 should be cited in this context. Previous evidence also points towards LTbR stimulation of FRC by DCs for podoplanin expression and pdpn-mediated cell survival (Kumar et al) which could be used to enrich the discussion.

We have included (additional) citations to *Yang et al. PNAS 2014* and *Kumar et al. Immunity 2015* in the introduction, results and discussion.

We have added the following sentences to the discussion to address the reviewer's points:

- Lines 393-395: "*Mechanisms controlling podoplanin expression in FRCs are incompletely understood. Upregulation and maintenance podoplanin expression may be mediated by LT□R stimulation by DCs (Kumar et al., 2015) and/or altered YAP/TAZ signalling (Choi et al., 2020).*"
- Lines 447-449: "*For example, podoplanin expression on FRCs is altered in lymph node- originated haematological malignancies ((Pandey et al., 2017; Apollonio et al., 2018) and own unpublished data).*"

2. The FRC subsets are oversimplified relative to the current literature. If TRC are considered the remaining fraction once MRC have been gated from the FRC population, then the use of this terminology should be explained in a sentence to state that this includes another major FRC subset of the medulla (Sitnik et al. Cell reports 2016, Huang et al., PNAS 2018; Rodda et al., Immunity 2018).

The reviewer correctly identifies that we have grouped together all FRC subsets residing in the T cell area and labelled them as TRCs (T-zone reticular cells). We have edited the text to make this clear to a broader readership not familiar with all the identified FRC subsets.

We have added the following sentence to the results (lines 134-136) to address this point: "*We here define TRCs to include all CD31-PDPN+MAdCAM-1- FRC subsets (Sitnik et al., 2016; Rodda et al., 2018; Huang et al., 2019).*", citing the three references above.

3. Figure 2 legend: please state on top or in each section that these are FRC lines investigated.

We have now included throughout the results of Figures 2-6 and in the corresponding figure legends that FRC cell lines have been investigated. The original immortalised FRC cell lines are characterised in the publication where they are first used (Acton *et al.* Nature 2014), and all knock out lines are generated from this polyclonal cell line.

© 2021. Published by The Company of Biologists under the terms of the Creative Commons Attribution License (https://creativecommons.org/licenses/by/4.0/).

4. Fig 2a/b: less surface expression can also be due to differences in translation or transport to the surface.

We thank the reviewer for bringing up this point. We agree that changes to podoplanin surface expression can be due to changes to translation and/or transport, in addition to the CLEC-2-dependent transcriptional increase of podoplanin expression.

In response to reviewer 2, comment 3, we have now switched panel a & b in Figure 2, which has resulted in stating our hypothesis/research question: "*We next asked if the changes in expression of podoplanin, CD44 and CD9 were transcriptionally regulated by CLEC-2.*" (lines 176-177).

Reviewer #1 (Significance (Required)):

These novel results, together with previous in vivo results, provide a deeper understanding of the process of lymph node swelling when the fibroblast network expands, highlighting some of the cellular processes and molecular players involved, and suggests new molecules to be studied in this process in vivo. This study should be of interest to people interested in FRC and lymph node swelling, or in the molecular processes of cell polarization, spreading and migration.

My expertise as well as the one of my co-reviewer (postdoc in my lab) are in lymph node FRC in development, homeostasis and immunity.
Reviewer #2 (Evidence, reproducibility and clarity (Required)):

de Winde and coworkers study in this work the events triggered by the binding of CLEC-2 to podoplanin on the surface of FRC cells. In vivo, this interaction is pivotal for lymph node expansion during the adaptive immune response since CLEC-2 on dendritic cells (DCs) inhibits podoplanin- induced actomyosin hypercontractility allowing FRC spreading. By using an immortalized FRC cell line either exposed to recombinant CLEC-2 or expressing CLEC-2-Fc (in order to mimick prolongued CLEC- 2 exposure from migratory DC cells arriving into the lymph node) and performing knockdown (shRNA) and KO (CRISPR/Cas9) experiments, the authors show that CLEC-2 upregulate podoplanin and CD44 expression at the transcriptional level. Podoplanin co-localizes with CD44 and its other partner CD9 in different regions of the plasma membrane, and both partners temper podoplanin- driven contractility. Podoplanin controls FRC cell motility and polarity while CD9 and CD44 control cell-cell interactions independently of podoplanin.

This is a well-written piece of work with interesting results showing an active role of podoplanin beyond CLEC-2-podoplanin-driven relaxation of actomyosin contractility, which in collaboration with CD44 and CD9 promote FRC protrusions and spread. However, there are some concerns that limit the scope and significance of the article:

We thank the reviewer for their comments which reflect a significant interest in podoplanin signaling in FRCs and other contexts.

1. All novel and significant results come from in vitro experiments using only a FRC cell line immortalized by infection with HPV-E6-encoding retrovirus. It is clear that investigating the roles of podoplanin, CD44 and CD9 on FRC expansion in vivo, in the context of the adaptive immune response, is extremely difficult and challenging. But at least two immortalized FRC cell lines should have been used in order to obtain more consistent results.

FRCs have been notorious difficult to immortalize, and so there are not a range of FRC cell lines to use in parallel. Earlier FRC studies had been limited to using either primary cells, which are not amenable to genetic modification, or *in vivo* observations until this cell line was generated. The FRC cell line (Acton *et al*. Nature 2014) used in this study has been generated by pooling FRCs ($CD45^-$, $CD31^-$, $PDPN^+$) from lymph nodes from multiple mice, and is maintained as a polyclonal cell line.
Furthermore, the CLEC-2-expressing, PDPN KD, PDPN KO, CD44 KO, CD9KO, CD44/CD9 DKO FRC cell lines used in our work are also all polyclonal cell lines. While we understand the point of the reviewer, in the case of this particular cell type, the models for *in vitro* experiments are very limited. We have here shown relevance of the function and coordinated expression of podoplanin,

© 2021. Published by The Company of Biologists under the terms of the Creative Commons Attribution License (https://creativecommons.org/licenses/by/4.0/).

CD44 and CD9 in FRC biology in multiple ways, which we believe convincingly supports our hypothesis. We agree that this limits the study to some degree, but the next steps to validate our findings would be to generate combinations of conditional deletions in mouse models which are not available at the current time.

2. We miss a proper characterization of knockdown and knockouts of the different molecules. These should be shown at least as supplementary data.

We agree with the reviewer that it would benefit the manuscript to include more details validating the newly-generated knockout FRC cell lines used for this study. We did not notice any obvious changes in viability and proliferation rate of KO cell lines used in this study. We have confirmed loss of podoplanin, CD44 and/or CD9 surface expression by flow cytometry as shown in Fig. 2d, and we have now included a supplementary figure (Figure S2) including qPCR and Western blot data of podoplanin, CD44 and CD9 expression in the KO cell lines used in this study.

PDPN KD cell line is generated and characterized previously (Acton *et al*. Nature 2014), which is referenced in the Methods, and we have now also included this reference in the results (line 182).

3. In Figure 2, where the expression of podoplanin, CD44 and CD9 are shown at the level of mRNA, protein or cell surface, only partial results are shown. Why not Western blots for CD44 and CD9 are presented in order to see changes in total protein expression?

We agree with the reviewer that the co-regulation of podoplanin, CD44 and CD9 is an interesting finding in our manuscript. As such, we have now included including analysis of total protein expression of CD44 and CD9 in Ctrl vs. CLEC-2-Fc FRCs (Figure 2, panel b). To improve the readability of our story, we have switched Figure 2, panels a & b to first show mRNA data (panel a) and then total (panel b) and surface (panel c) protein expression.

4. It is shown in Figure 2 that PDPN knockdown reduces CD44 and CD9 mRNA and cell-surface expression. On the other hand, FRCs KO for CD44 and CD9 showed reduced cell-surface PDPN expression, revealing interdependence in the expression of these proteins. But, does silencing of CD44 and CD9 affect PDPN mRNA and protein expression? It should be important to unveil whether a reciprocal regulation in the expression of these proteins exist.

We agree, and as detailed above (comment 2), we have included a supplementary figure (Figure S2) including qPCR and Western blot data of podoplanin, CD44 and CD9 expression in the KO cell lines used in this study.

5. How co-localization is measured in Figure 2e in single sections or in the maximum Z stack projections shown? In general, the immunofluorescence analysis reported is deficient and not clear. It seems to contain a high degree of unspecificity.
Figure 2e shows co-localization of PDPN with CD44 and CD9 in FRC control and CLEC-2-Fc-expressing cells, but does CD9 co-localize with CD44? Did the authors explore the existence of a ternary complex? In this respect, authors assume that co-localization is equivalent to interaction, and this might not be true.

We apologies for this omission, the data in Figure 2e represents Pearson's coefficient analyzed in single sections using JaCoP plugin in Fiji/ImageJ. We have now included more detailed information in the Methods (lines 601-603) and corresponding Figure legend (lines 882-883).

Regarding the second point exploring the possibility of a ternary complex, a question also raised by reviewer 1 (comment 5), we have now included analysis of colocalization of CD44 and CD9 to extend Figure 2 (panel f), presenting examples of triple stained cells in steady state and in the presence of CLEC-2.

The reviewer is also correct that we cannot assume that colocalization is equivalent to interaction, and since we show confocal images, the resolution is not sufficient to show complexes. However, our intention with these data is to show firstly that CD9 and CD44 both colocalize with podoplanin in different regions of the cell membrane, and secondly to ask whether

© 2021. Published by The Company of Biologists under the terms of the Creative Commons Attribution License (https://creativecommons.org/licenses/by/4.0/).

colocalization is altered in the presence of CLEC-2. We do not mean to imply that colocalization is equivalent to molecular interaction/binding, and have amended the text accordingly.

6. In Figure 3, contractility of CD44/CD9 double KO FRCs is highly reduced upon expressing CLEC-2, what allows the authors to conclude that hypercontractility in the double KO is podoplanin-dependent. Although podoplanin is the only natural ligand of CLEC-2, this assertion should be confirmed by silencing podoplanin expression. It should be taken into account that the double KO of CD44/CD9 reduces cell surface podoplanin expression (Fig. 2d).

Although we have not generated a triple knockout cell line, we can address this important point in two ways.

Firstly, it is previously published that CLEC-2 deleted dendritic cells are unable to induce FRC spreading (de Winde *et al.* J Cell Sci 2018), and in the current manuscript (Figure 6a-b) we highlight to the reader how dendritic cell morphology does not change in co-culture with podoplanin-deficient FRCs (lines 334-335).

Secondly, we have included additional data comparing the morphology of control and podoplanin KO FRCs expressing CLEC-2 (Figure S3). Here, we observe that CLEC-2 has no effect on FRC morphology in the absence of podoplanin (lines 249-251).

7. In Figure 4a and b, the way migration parameters are presented are somehow confusing. I cannot understand what coloured circles represent in panel b.

We have changed the figure legend to clarify what the coloured circles represent, which now reads (lines 920-921): "*The intensity of the coloured circles represents the number of cells moving the same distance from the starting position.*"

We have also added an additional explanation to the Methods section (lines 626-629): "*To show the maximum distance travelled by cells, overlaid semitransparent circles were plotted with radii equivalent to the maximum distance travelled by each cell from the origin (calculated as $r = \max\left(\sqrt{(x-x_0)^{2}+(y-y_0)^{2}} \right)$).*"

8. In Figure 5, the authors state an increase of ARPC2 staining in membrane protrusions of FRCs lacking PDPN, CD44 or CD9, which I do not see so clearly in the pictures shown. Presence of ARPC2 on membrane protrusions should be quantified by some mean in a significant number of cells.

We agree that quantification of these data would strengthen this figure. We have now quantified ARPC2 expression on membrane protrusions of FRC cell lines by measuring mean fluorescence intensity (MFI) on the cell membrane as a ratio of MFI in the cytoplasm using line plots (Figure 5a-b), as detailed in the Results (lines 311-313) and Methods (lines 607-609).

9. Since CLEC-2 binding inhibits podoplanin-dependent activation of RhoA/RhoC signaling activity, which are the signaling events associated with FRC spreading and elongation? The lack of this type of studies make this paper somehow incomplete.

This is a very broad question, and reflects the fact that signaling downstream of podoplanin is still not well understood. Our data present additional roles of molecular players in addition to podoplanin, but we have not overstated our findings, and simply show that inhibition of RhoA/C is just one part of the complex response of FRCs to dendritic cells. The signaling events associated with FRC spreading include activation of Rac1 (Acton *et al.* Nature 2014), but now requires further investigation in the context of CD44 and CD9.

Minor points:

-Authors state referring the published work of another group that the interaction of podoplanin with CD44 is mediated by the extracellular domains (ref. 19), although it was recently reported that this interaction also involves the transmembrane and cytosolic regions (ref. 20). This sentence is wrong, or at least confusing, since in the latter reference the above mentioned group

© 2021. Published by The Company of Biologists under the terms of the Creative Commons Attribution License (https://creativecommons.org/licenses/by/4.0/).

retracted their former conclusion, showing that PDPN-CD44 interaction is mainly mediated by the transmembrane domains, with the cytosolic and extracellular regions modulating this interaction.

We apologize for this incorrect citation, and have now changed this information in our manuscript (lines 96-98): "*Podoplanin interacts with CD44 through their transmembrane domains, and this interaction is modulated by both the cytosolic and transmembrane regions (Montero-Montero et al., 2020).*" as well as in lines 201-202.

-In page 5, FRCs moving at speeds <0.2 ?/min (□m?)

Sorry for the confusion, this is a typo. It is indeed □m, and we have now changed this is the text (line 278).

Reviewer #2 (Significance (Required)):

This paper continues a series of studies performed by the group focused to understand the role of podoplanin in the remodelling of lymph nodes during the adaptive immune response. In FRCs, podoplanin promotes actomyosin contractility through its binding to ERM proteins and activation of RhoA/C GTPase, and binding of CLEC-2 located on migratory DCs inhibits podoplanin-induced contractility, resulting in FRC spreading and elongation to allow a rapid lymph node expansion. In a previous report, de Winde and colleagues suggested that upon binding of CLEC-2 to FRCs, podoplanin is recruited to cholesterol-rich domains (lipid rafts) where interacts with membrane partners CD44 and CD9 to form cell protrusions and to spread, issues that have studied in the present paper.
While the results obtained are relevant for those readers interested in understanding the role of podoplanin in lymph node remodelling or cell motility, this work is weakened by several facts: i) results come from only a FRC cell line; ii) no interaction of podoplanin with CD44 and CD9 is shown, but only immunofluorescence co-localization; iii) no signaling events downstream the CLEC-2- podoplanin axis associated with FRC spreading and elongation have been studied.

We thank the reviewer for their constructive comments.

Reviewer #3 (Evidence, reproducibility and clarity (Required)):

The authors in this manuscript are investigating the mechanisms by which Clec-2/podoplanin interactions between dendritic cells (DCs) and fibroblastic reticular cells (FRCs) facilitate FRC spreading and lymph node (LN) expansion. This paper makes a significant contribution to understanding how expansion of the FRC network in LNs is accomplished during an infection by DCs. Previous studies have established the critical role of CLEC2 on DCs in LN expansion. What is not understood is how CLEC2 binding to FRCs facilitates their expansion and how podoplanin interacts with its partners in FRCs to accomplish this in response to DCs.

In the first two studies presented, the authors find that 1) podoplanin and its partner proteins CD44 and CD9 are coregulated in FRCs by Clec2, 2) podoplanin, CD44, and CD9 are differentially expressed in different LN stromal populations, and 3) CD44 and CD9 co-localize with podoplanin in different areas of the cell membrane. Moreover, they show that CD44/podoplanin complexes are induced by CLEC2, while CD9/podoplanin complexes are not. They next explored the extent to which CD44 or CD9 control different aspects of FRC function, including contractility, migration, alignment, cell-cell network interactions, and membrane protrusions. Here they find that CD44 is required for contractility, suppression of protrusions, suppression of migration, and enhances cell-cell interactions; while CD9 suppresses protrusions, migration, and cell-cell interactions, with some effects being independent of podoplanin. Neither is required for alignment. These studies nicely define different roles of podoplanin, CD44, and CD9 in FRCs.

However, there is a major concern that some of the studies appear to be have been conducted on FRC cells without any exposure to Clec2, while others included Clec2. Thus, it is hard to distinguish specifically what functions of FRC studied are simply intrinsic to the FRC independent of Clec2 versus those that are specifically controlled by Clec2. These creates some confusion in their conclusions.

© 2021. Published by The Company of Biologists under the terms of the Creative Commons Attribution License (https://creativecommons.org/licenses/by/4.0/).

We thank the reviewer for their positive review. We agree that where some data are limited to the steady state functions of podoplanin, CD44 and CD9 in FRCs, the manuscript would be strengthened by comparing these phenotypes in the presence of CLEC-2 (in particular in figure 4 and 5).

Additional points to address include:

1) Concluding paragraph for Figure 2 data near the end of page 3 is not quite correct with respect to what is happening with CD9. In addition, to support their conclusion that CD9/PDPN and CD44/PDPN complexes maybe different, colocalization experiments with CD9/CD44 should be done.

As also raised by reviewers 1 and 2, we have now included triple staining of podoplanin, CD44 and CD9 in steady state and in the presence of CLEC-2, and colocalization of CD44 and CD9 (Figure 2, panel f). These data indicates the possibility of a ternary complex of podoplanin and its partners proteins CD44 and CD9 on the plasma membrane of FRCs.

2) No stats are provided for the graphs in Fig 2c and 2d.

We have now added statistical significance or “ns” to Figure 2, panels c-d, and all other appropriate figure panels.

3) In Fig. 3, Knock-down of both CD9 and CD44 together are required to reverse the contractility induced by Pdpn, while overexpression of either are sufficient to rescue contractility. Yet, only the CD44/pdpn complexes are altered upon Clec-2 binding. Thus, it is not clear how biologically important CD9 is to the process of contractility in response to Clec2? What happens to FRC expansion/function in CD9 knock out mice?

The reviewer raises a valid point which we do not fully address with the data as presented. It is possible that over-expression of CD9 rescues hypercontractility because the levels of expression are higher than the endogenous CD9. Since these data are ambiguous, we have added the following sentences to the discussion to reflect the still open question of the role of CD9 in FRC contractility: “*However, in presence of CLEC-2, which inhibits podoplanin-driven contractility, co-localization of podoplanin with CD9 remains unaltered in contrast to podoplanin/CD44 co-localization. Thus, the exact role of CD9 in controlling FRC contractility and the molecular mechanisms involved requires further investigation.*” (lines 415-418).

The reviewer is correct, that to understand the role of CD9 in FRC function fully, we would need to look *in vivo*. However, CD9 is broadly expressed, including on other cells in the lymph node. Full CD9 KO mice are infertile (due to the pivotal role of CD9 in sperm-egg fusion; Kaji *et al.* Nat Gen 2000), which creates an extra hurdle to make conditional CD9 KO mice. Indeed, CD9 expression on T cells is required for efficient co-stimulation (Tai *et al.* J Exp Med 1996), meaning that antigen-presentation and T cell proliferation would be attenuated in CD9 KO mice, and therefore lymph nodes would not expand to the same degree as controls. As such, to investigate the *in vivo* role of CD9 on FRC expansion/function, CD9 needs to be conditionally knocked-out on FRCs, which is not currently possible.

4) An important conclusion from the data in Fig. 4 is that CD44 and CD9 each independently limit baseline FRC motility, but have no impact on alignment. How do these cells respond to motility and alignment when subjected to rCLEC2? Ditto for Fig. 5, how are FRC-FRC interactions impacted by exposure to Clec2. These studies would be much stronger and more robust conclusions drawn about their role in Clec2-dependent processes if this is investigated.

We agree that the manuscript would be strengthened if we include both steady state and CLEC-2 stimulation. We have now included analysis of CLEC-2-expressing FRC cell lines missing podoplanin, CD44 and/or CD9 expression to Figures 4 (panel c) and 5 (panels c-d), and discussed these data in the text (respectively lines 298-300 and 318-322).

5) The data in Fig. 6 is very strong, as it the most biologically relevant and really highlights the dependency on both CD9 and CD44 for the morphological response.

© 2021. Published by The Company of Biologists under the terms of the Creative Commons Attribution License (https://creativecommons.org/licenses/by/4.0/).

We thank the reviewer for this positive assessment. These data represent our strongest evidence that FRCs require CD44 and CD9 in addition to podoplanin to respond to dendritic cells.

Reviewer #3 (Significance (Required)):

This paper makes a significant contribution to understanding how expansion of the FRC network in LNs is accomplished during an infection by DCs. Previous studies have established the critical role of CLEC2 on DCs in LN expansion. What is not understood is how CLEC2 binding to FRCs facilitates their expansion and how podoplanin interacts with its partners in FRCs to accomplish this in response to DCs. These studies nicely define different roles of podoplanin, CD44, and CD9 in FRCs.
Audience: Those interested in lymphocyte/immune cell biology, cell-cell signaling mechanisms, tetraspanins and associated proteins,

My expertise: tetraspanins, signaling, and motility

Second decision letter

MS ID#: JOCES/2021/258610

MS TITLE: Fibroblastic Reticular Cell Response to Dendritic Cells Requires Coordinated Activity of Podoplanin, CD44 and CD9

AUTHORS: Charlotte M de Winde, Spyridon Makris, Lindsey Millward, Jesús Cantoral, Agnesska C Benjamin, Victor G Martinez, and Sophie Acton
ARTICLE TYPE: Research Article

Thank you for sending your manuscript to Journal of Cell Science through Review Commons. I am happy to tell you that your manuscript has been accepted for publication in Journal of Cell Science, pending standard ethics checks.

© 2021. Published by The Company of Biologists under the terms of the Creative Commons Attribution License (https://creativecommons.org/licenses/by/4.0/).
